# Application of fractional-order synergetic-proportional integral controller based on PSO algorithm to improve the output power of the wind turbine power system

**DOI:** 10.1038/s41598-024-51156-x

**Published:** 2024-01-05

**Authors:** Habib Benbouhenni, Gasmi Hamza, Mihai Oproescu, Nicu Bizon, Phatiphat Thounthong, Ilhami Colak

**Affiliations:** 1 Department of Electrical and Electronics Engineering, Faculty of Engineering and Architecture, Nisantasi University, 34481742 Istanbul, Turkey; 2 LaboratoireControleAvancé (LABCAV), Department of Electronics and Telecommunications, Université 8 Mai 1945 Guelma, BP 401, 24000 Guelma, Algeria; 3 The National University of Science and Technology POLITEHNICA Bucharest, Pitești University Center, 110040 Pitesti, Romania; 4 ICSI Energy, National Research and Development Institute for Cryogenic and Isotopic Technologies, 240050 Ramnicu Valcea, Romania; 5 Group of Research in Electrical Engineering of Nancy (GREEN), University of Lorraine-GREEN, 54052 Nancy, France; 6 Renewable Energy Research Centre (RERC), Faculty of Technical Education, King Mongkut’s University of Technology North Bangkok, 1518 Pracharat 1 Road, Wongsawang, Bangsue, Bangkok, 10800 Thailand

**Keywords:** Energy science and technology, Engineering

## Abstract

It is noted that the traditional direct filed-oriented control (DFOC) is widely used in the field of electric power generation from wind due to its fast response dynamic, ease of implementation and simplicity, but this strategy is characterized by the presence of large ripples at the level of both active and reactive powers. This work presents a new algorithm for DFOC strategy of an asynchronous generator (AG) in a wind power (WP) system, which is based on the use of a new nonlinear controller called fractional-order synergetic control–fractional-order proportional-integral (FOSC–FOPI) controller, where the proposed technique parameters are calculated using the particle swarm optimization (PSO) strategy. It has been observed that the DFOC–FOSC–FOPI–PSO strategy is robust and works well in case of changing generator parameters. Three tests were performed to study the behavior of the designed technique under different working conditions, where the behavior of the DFOC–FOSC–FOPI–PSO strategy was compared with the behavior of the traditional DFOC technique in terms of power ripple ratio, overshoot, steady-state error, response time, tracking reference, and current quality. The simulation of the designed technique based on the FOSC–FOPI–PSO strategy of the AG–WP system is carried out using Matlab software, where the simulation results showed that the suggested technique is better than the classical technique (with PI controller) in terms of improving response time of active power (33.33%) and reactive power (10%) in second test, reduction of the steady-state error of reactive power (96.95%) and active power (97.14) in first test, minimization of harmonic distortion of current (96.57%) in third test and significant minimization of ripples of active power (99.67%, 44.69%, and 98.95%) and reactive power (99.25%, 53.65%, and 70.50%) in the three tests. The effectiveness of the DFOC–FOSC–FOPI–PSO strategy is very high, so it can be a reliable solution for controlling various generators.

## Introduction

In the other two decades, there has been a noticeable decrease in the consumption of fossil fuels to produce electrical energy, and this traditional energy has been replaced by other sources under the name of renewable energy (RE), as this decrease is shown by the proportions of fossil fuel consumption in the world from 86% in 1973 to 81% in 2016^1^. In addition, the widespread and increasing use of REs in the production of electrical power, such as solar energy and wind power (WP). WP is a branch of RE, which is clean, non-polluting energy that is used to generate electric power from wind all over the world. Also, the use of the wind area contributes significantly to reducing CO_2_ emissions and contributes significantly to reducing the production bill and traditional energy consumption^2^.

In wind stations, wind is used to obtain mechanical energy through a wind turbine (WT) system that consists of three main components: an electric generator, a WT, and a gear^3^. Electric generators are used to convert the mechanical power gained from the wind into electrical power, where the power electronics are used to transfer this generated energy to the grid at a frequency of 50 Hz^4^. There is a noticeable development in recent years in WP generation systems, which have become less expensive and smaller due to the rapid development of power transformers^1^. In addition, there is an improvement in the power generation capacity of WT systems due to an increase in the rotor size of WTs and the emergence of other more efficient types of WTs^5^. In^6^, the author proves that the power generated by the WT system can be controlled by increasing the diameter of the turbine rotor. In^7^, a multi-rotor WT (MRWT) was used to generate electricity from wind. The generation system is considered among the most complex systems, as there are several factors that control the generation of electrical energy from wind^8^. Among these factors can be mentioned wind speed (WS), external meteorological conditions, non-linear internal dynamics, unknown turbulence, and parametric uncertainties^7,8^. To transmit electrical power to the grid a control system is necessary, as there are several different types of electrical and mechanical control schemes that have been used in the generation system^9,10^.

In the field of WT system, algorithms are used to obtain the maximum energy extraction. These algorithms are known as the maximum power point tracking (MPPT) algorithms^11^. The latter is only used for variable-speed WT systems (VSWTSs) using a energy electronics transformer in full or partial arrangement^11^. This method is simple and depends on the use of a proportional-integral (PI) controller^12^. This strategy is of great importance in the field of RE, especially WP, as this strategy is used to obtain the reference value for active power (*P*_*s*_), which makes the resulting energy related to the WS, which is a good thing. But using the MPPT technique based on the PI controller gives less effectiveness, as it is known that the PI controller is less efficient in the event of a malfunction and its use leads to ripples at the coefficient of power. The latter is of great importance in calculating the energy gained from wind. In addition, ripples cause poor operation and lead to regular maintenance, thus increasing costs, which is undesirable. So, the MPPT method is developed using new techniques such as artificial intelligence and nonlinear methods to improve the maximum energy extraction^13^.

As is known, generating electrical energy depends on the principle of converting mechanical energy into electrical energy using electrical machines called generators, where the resulting electrical energy depends on the capacity of the generator. In addition, the power of the generator is related to the power of the turbine, as it is not possible to use a generator with a power greater than the power of the turbine. Therefore, the generator and turbine are used with the same capacity to obtain good operation of the system and to reduce periodic maintenance.

In a wind system, there are three main types of electrical generators that can be used to generate electrical power such as DC generators, synchronous generators (SGs), and asynchronous generators (AGs)^9,10^. Also, other generators can be used in a wind generation system such as squirrel cage induction generators (SCIGs)^14^, wound rotor induction generators (WRIGs)^15^, frequency starter generators (SRGs)^16^, variable frequency generators (VRGs)^17^, and synchronous permanent magnet generators (PMSGs)^18^. In addition, DC generators are not used to generate electric power due to their high cost and frequent and meticulous maintenance^1^, where these generators are used in the case of small turbines along with batteries. On the other hand, the electrical machines have pros and cons, as using these machines requires the use of an inverter to supply them and control the resulting energy. In the case of synchronous machines, an inverter is used that is connected to the stator of the machine. But in the case of asynchronous machines, two reflectors are used, which are connected in a head-to-head shape and are attached to the rotating part of the machine. The speed of rotation of the machine can be controlled using these two reflectors, which gives an advantage to these machines over synchronous machines.

### Literature review

Traditionally, electrical machines are machines that can operate motors or generators, depending on the energy used to feed them. If the electrical energy is what feeds the machine in this case, the machine is a motor, but if the mechanical energy is what is used to feed the machine in this case. The machine is a generator. So, in the field of REs, especially in WP, the machine is operated according to the WS, avoiding operating the machine in an engine state. In this case, the machine consumes energy, which is undesirable. Therefore, protection is used when the wind is strong, as well as when it is weak, to avoid consuming energy without purpose.

AGs are among the most popular and widely used generators in the field of WT system, as they are the dynamic slip-controlled generator^9^. This generator is characterized by ease of control, high durability, low cost, low maintenance, and the output power can be controlled by feeding the rotor, as this is one of the most prominent features that make it the most used generator in the case of variable WS. To feed the AG from the grid, AC–DC–AC transformers are used^13,19^. These inverters have the advantages of high quality and smooth transmission of electricity after extracting maximum power from the wind^2^. Also, these inverters are used to control AG rotor speed by excitation of alternating current and perform separate control of *P*_*s*_ and reactive power (*Q*_*s*_) with *Q*_*s*_ compensation^13^. To operate the inverter, a signal in the form of pulses is used. These pulses are used to turn on and off the transistors that make up the inverter. Therefore, several strategies can be used to generate these pulses. Pulse width modulation (PWM) and space vector modulation (SVM) are among the most prominent and most widely used strategies to control the operation of an inverter. The PWM technique is simple, easy to perform, and inexpensive compared to the SVM technique. But the SVM technique is more efficient and performant than the PWM strategy in terms of reducing the current ripples coming out of the inverter and reducing the value of total harmonic distortion (THD) of voltage.

The problems of a generation system based on AG are protection problems under network fault disturbances and the use of slip rings^20^. AGs are used for offshore wind farms where electricity is transmitted using high-voltage direct current (HVDC) transmission lines^21^. Thyristors are used in transformers to reduce mechanical stress and limit currents flowing.

The AGs is among the most widely used and most suitable generators in the case of VSWTS, as this generator is suitable for generating electric power from wind with variable speed compared to several generators such as DC generators^22^. Also, the AG can be easily controlled using several controls, for example direct torque control (DTC)^23^, field-oriented control (FOC)^24^, vector control (VC)^25^, backstepping control (BC)^26^, synergetic control (SC)^27^, direct power control (DPC)^28^, sliding mode control (SMC)^29^, passivity control^30^, high-order SMC (HOSMC) technique^31^, and fractional-order control^32^. Some of the strategies mentioned above depend on the use of a switching table (ST) to generate control pulses in the inverter, where a hysteresis comparator (HC) is used to obtain ST inputs such as DPC and DTC. There are also strategies that rely on the use of SVM or PWM to generate the pulses necessary to operate the inverter, where a PI controller is used for the purpose of determining reference voltage values, such as VC technique. Two strategies, vec VC technique and FOC technique, can be found among the most prominent strategies that rely on the use of the PI controller to control the characteristic quantities. FOC strategy has been used in control in recent years as the most suitable solution, as there are two strategies: the direct FOC (DFOC) strategy and the indirect FOC (IFOC) strategy. The difference between these two strategies lies in the number of PI controllers used.

In general, these strategies can be divided into four different families, where there are families of linear controls such as DPC and DTC, where the linear controls are among the most famous of these strategies because of the ease of implementation, simplicity and speed of great dynamism that characterize them. However, these strategies depend on the estimation of each of the *P*_*s*_ and *Q*_*s*_, which is undesirable, as the estimation of the capabilities makes the system less efficient in the case of changing the machine parameters. A family of nonlinear controls such as SMC and SC technique, where the nonlinear strategies are better compared to linear strategies in terms of robustness. However, these strategies have an undesirable advantage of complexity, as they are difficult to apply to systems, especially complex ones such as SMC and BC techniques. Moreover, these strategies are highly dependent on the system parameters which is undesirable, which creates many problems in case of machine failure. A family of intelligent controls such as neural networks (NNs) and fuzzy logic (FL) control, where these strategies are controls that do not require precise knowledge of the mathematical model of the system, are inexpensive, simple, depend on experience in the application, and can be easily accomplished. Also, it has good dynamic speed, which makes it a suitable solution in the field of control. In addition, in the case of changing the parameters of the machine, it gives excellent results, as it is not affected by this change, as is the case in the case of linear and non-linear strategies. Finally, hybrid control that relies on integrating between different or similar controls such as SC–SMC technique, neuro-SMC technique, and neuro-fuzzy control. Hybrid control techniques that appeared as an alternative to other strategies are characterized by high performance and greater efficiency than other techniques. These control techniques depend on the combination of two different control techniques, such as the combination of DPC and SC technique, or similar ones, such as a combination of FL and genetic algorithm, where the combination leads to significantly improving the characteristics of the control techniques while minimizing system defects such as power ripples and THD of current. Sometimes the fusion leads to obtaining a new but much more complex technique, and this is not desirable, as in the case of the merger between SMC technique and BC technique, where the complexity leads to the difficulty of implementation, an increase in the cost of the system, an increase in energy consumption, and it makes maintenance complicated. All of these control families were used to control AG-based WT system, which provided satisfactory results to some extent. However, the problem of energy ripples and the quality of the current remains one of the most prominent problems for these control techniques.

In contrast, DPC uses HCs to control the AG power and thus is simpler, easier and less expensive to perform compared to the FOC strategy^31^. The DPC strategy is very similar to the DTC, but in the DPC strategy the *P*_*s*_ and *Q*_*s*_ is controlled directly using a ST^28^. In the DPC strategy, both *P*_*s*_ and *Q*_*s*_ are controlled without the use of internal loops^33^. This strategy was used to control several generators such as SGs and induction generators^34^. Several papers highlight the disadvantages of the DPC strategy, and *P*_*s*_ and *Q*_*s*_ ripples are among the major drawbacks of this technique^28,33^. In addition to the high value of THD of current, which indicates that the quality of the current is low, this matter is considered undesirable, causing several defects and problems and increasing regular maintenance and thus costs.

In traditional DPC technique, the quality of the *Q*_*s*_/*P*_*s*_ is lower due to the use of HCs. Its reliance on a ST to control the inverter, along with its use of simple controls, makes it one of the easiest and least expensive strategies compared to other techniques such as BC technique or indirect FOC technique^35^. The reliance of this strategy on estimating both *P*_*s*_ and *Q*_*s*_ makes the system affected in the event of a malfunction in the machine, as the power estimate is related to the stator resistance (*R*_*s*_), and this appears clearly in the significant increase in power ripples and the value of THD of current in the durability test performed in scientific works^33,34^. To tackle these disadvantages, various strategies have been used in the literature such as PI controller^36^, SMC technique^37^, and FL technique^38^. Most of these proposed solutions depend on removing both the ST and two HCs and replacing them with other strategies to improve performance and increase the efficiency of the DPC strategy.

In^39^ a new strategy for DPC was designed, whereby the ST and HC loops were dispensed with and instead both PWM technique and HOSMC controller were used. A HOSMC controller is done to calculate the reference values for rotor voltage. The latter is used to calculate the three-phase voltages of the PWM technique. Matlab software was used to implement the proposed nonlinear strategy and compare it with the traditional control. The simulation results showed the superiority of the HOSMC controller over the traditional controller in terms of reducing ripples and improving the quality of the current. Using a HOSMC controller increases the complexity slightly, as there are a significant number of gains, which makes it difficult to adjust the dynamic response and use artificial intelligence strategies to determine the values of these gains. In addition to using power estimation, which increases the rate of ripples if the machine parameters change, which is undesirable. A robust DPC strategy was designed in^40^, where the *P*_*s*_ and *Q*_*s*_ of generator was regulated using BC technique and then appropriate PWM signal is commanded. The use of a BC technique increases the complexity of the DPC strategy, as there are a significant number of gains, which makes it difficult to adjust the response. In addition, the DPC strategy becomes related to the mathematical model of the machine, which makes the proposed strategy affected in the event of a malfunction in the system, such as changing parameter values, which is undesirable. The use of a BC technique makes the DPC strategy more complex, difficult to implement, and expensive. But the simulation results proved that the use of a BC technique led to a significant improvement in the characteristics of the generation system while increasing its durability in the event of a change in the machine parameters. Despite this positivity, the problem of energy ripples remains present in this strategy. A neural DPC strategy was proposed in^41^, where the effectiveness of AG in different tests were investigated. NNs technique were used to compensate for the ST. Using this strategy in generating control pulses helps increase control robustness, performance, and efficiency in reducing the value of THD of current compared to the DPC technique. The negative of the proposed control is the use of the power estimation process, which makes the control dependent on the machine’s parameters, which makes the system affected if the parameters change, which is undesirable and helps to significantly reduce the power quality. However, the use of NNs technique gave very satisfactory results and contributed to reducing energy ripples and significantly improving the dynamic response. As is known, the disadvantage of controlling the use of NNs technique is the lack of mathematical rules that help in choosing the number of internal layers and neurons, as it depends largely on experience. Therefore, several solutions must be applied, and then the best solution is chosen from among several solutions, which makes the matter somewhat complicated. A DPC–SMC control for AG-based WTS was addressed in^42^, where minimizing the ripples of current and *Q*_*s*_ was the main aim of this work. The DPC–SMC strategy is radically different from the traditional strategy, as the two HCs and ST are eliminated and replaced by the SMC controller and SVM technique compared with the traditional strategy, DPC–SMC control is more complex, expensive, and difficult to implement. The DPC–SMC strategy has a similarity to traditional control, which is the use of the same estimation equations. Matlab software was used to implement the DPC–SMC strategy and compare it with the traditional control. The results showed high performance and superiority of the DPC–SMC control over the traditional control in the durability test and other tests. In^43^, the behavior of AG under different tests was studied and a intelligent DPC strategy was designed as being appropriate to improve current quality. The neural PI controllers were used together with the modified SVM (MSVM) to increase the quality of the *P*_*s*_ and reduce the current and torque fluctuations in this strategy. In this strategy, NNs were used to compensate for the PI controllers used to control *P*_*s*_ and *Q*_*s*_, where the outputs of the NNs technique are reference voltage values. The latter is used by the MSVM strategy to generate the pulses necessary to operate the generating inverter. The use of a neural PI controller led to a significant improvement in the DPC strategy, and this is demonstrated by the results obtained compared to the DPC strategy. A nonlinear DPC technique for AG was presented in^44^, where MRWT system is used to convert WP into mechanical power. This designed DPC technique is simple and robust compared to several techniques such as DPC and indirect FOC strategies. Matlab software was used to implement the proposed control and compare it with the traditional strategy in terms of THD value of current, power ripples, tracking references, and in terms of robustness. It is noticed that the ripples in the *P*_*s*_/*Q*_*s*_ and current are greatly minimized compared to the DPC strategy. A new zero DPC technique for *P*_*s*_ and *Q*_*s*_ compensation of harmonics using shunt *P*_*s*_ filter^45^. Zero DPC technique is a new strategy different from the traditional strategy, as it relies on power estimation, which makes measuring voltage and current necessary. Experimental work was used to verify the efficiency of the proposed zero DPC technique. The experimental results obtained were compared with other modern strategies to confirm the robustness and high performance of the designed zero DPC technique. Despite the results presented, the problem of power ripples and current quality remains present, which makes the search for the best strategy ongoing. An integral SMC (ISMC) technique was suggested to improve the performance of three-level neutral-point-clamped inverter^46^. DPC based on ISMC strategy differs from traditional control in terms of the controllers used to control the power and to control the inverter, as the SVM technique is used in this proposed strategy for the purpose of controlling the generating inverter. So the DPC based on ISMC technique is a complex and difficult strategy to implement compared to the DPC strategy, which is an undesirable matter that makes the generation system expensive and requires periodic maintenance. In addition, the use of the ISMC strategy makes control related to the mathematical model of the machine, which creates several problems and defects in the event of a malfunction in the machine, which is a problem. The DPC based on ISMC strategy was confirmed experimentally and the results were compared with traditional control in terms of performance and effectiveness in several different tests. Experimental results show the superiority and characteristic of the DPC based on ISMC strategy compared with DPC-PI technique. Another DPC strategy based on parallel model predictive control was used to improve the efficiency of the DPC strategy for WP conversion is proposed in^47^. DPC strategy based on parallel model predictive control is a strategy based on prediction, which is fundamentally different from traditional control. The advantage of this strategy is that it is robust, but it is characterized by complexity and the presence of a large number of parameters. In addition to the use of the mathematical model of the machine to a large extent, which makes it affected in the event of a malfunction in the machine. Matlab software was used to verify this strategy and study its behavior compared to the DPC technique. Despite the complexity, a significant number of gains, and their connection to the mathematical model of the machine, this strategy provided very satisfactory results in all tests, especially in the case of the durability test compared to the DPC technique. In^48^, SMC and SC technique are combined to overcome the flaws of the DPC strategy of AG-based MRWT system. In this strategy, the combination between SMC and SC technique was used as a suitable solution to improve the performance of the DPC strategy of AG-based MRWT, where the proposed controller (SC-SMC) differs radically from both SC technique and SMC in terms of complexity, simplicity, number of gains, ease of application, ease of implementation, performance, and durability. Two SC–SMC controllers were used to control AG power, with the PWM strategy used to control the AG inverter. The DPC–PWM strategy based on SC–SMC controllers was verified for its effectiveness and efficiency using Matlab software on a 1.5 MW AG-based MRWT system, where the results showed the distinctive performance of this strategy in terms of reducing ripple rates, response time, THD value of current, overshoot, and steady-state error (SSE) of *P*_*s*_ and *Q*_*s*_ compared to the traditional DPC technique based on HCs. A passivity DPC technique was presented in^49^, where the PWM technique is used to control the shunt *P*_*s*_ filter. The passivity DPC strategy is more complex than the DPC technique. But this technique has a higher efficiency in improving the quality of the current compared to the DPC technique. In^50^ a DPC technique based on neuro-fuzzy algorithms was designed to control the RSC of a AG-WT system, where the proposed DPC strategy is studied in the case of changing and not changing the parameters of the system. Two neuro-fuzzy controllers were used for the purpose of increasing the efficiency and performance of the DPC strategy, as these controllers were used in place of traditional controllers. In addition to using the SVM technique for the purpose of controlling the inverter. The advantage of this strategy is high durability and outstanding performance compared to the traditional strategy. However, this strategy has drawbacks, namely the estimation of capabilities, the lack of mathematical rules for choosing the rules of FL technique, and the number of internal layers/neurons of NNs necessary for the purpose of forming the proposed controller. This strategy depends heavily on experience, which makes performance dependent on the extent of experience in choosing the number of rules and the number of neurons needed. In^51^, a modified super-twisting algorithm (MSTA) which is capable of minimizing the *P*_*s*_ and *Q*_*s*_ ripples of the AG–WT system using PWM technique is proposed. In addition, DPC technique was designed for controlling the switched reluctance generator, whereby fractional-order PI (FOPI) controller is used to increase generator efficiency and improve power quality^52^. The proposed FOPI controller was optimized by harmony search optimisation algorithm to obtain a good result compared to the DPC technique. Through the experimental results obtained in^52^, the DPC–FOPI strategy is much better than the DPC-PI technique in terms of minimizing the *P*_*s*_/*Q*_*s*_ ripples and SSE value for both the *Q*_*s*_ and *P*_*s*_. In^53^ a novel DPC technique based on particle swarm optimization (PSO) algorithms was proposed to reduces the *P*_*s*_/*Q*_*s*_ and current ripples. The DPC–PSO strategy is a robust, simple, and gives better results for THD value, and improve the response dynamic compared to DPC technique. In this strategy, the PSO technique was used to calculate the controller parameters PI of *P*_*s*_ and *Q*_*s*_. This strategy has the disadvantage of estimating powers, which gives unsatisfactory results in the durability test in terms of current quality and power ripples. In addition to using the SVM strategy for the purpose of controlling the inverter, it makes the control somewhat complicated and expensive, and thus will increase the final cost of the energy produced, which is of course undesirable. In^54^, an improved DPC strategy based on VC technique was proposed to overcome the disadvantages of the traditional DPC technique. The use of the VC technique to improve performance makes the DPC control linked to the mathematical model of the machine with a significant number of gains, which makes adjusting the dynamic response difficult. In addition to the complexity that characterizes the strategy, DPC based on VC technique compared to the DPC technique. Despite all this, the proposed strategy based on VC technique provided satisfactory results in terms of reducing energy ripples. A modified DPC strategy based on best fuzzy-switching state approaches was presented in^55^. In this strategy, the ST is compensated by FL technique to generate the necessary pulses and obtain outstanding performance. However, this modified DPC technique was used to control the 3-phase PWM rectifier, where the robustness is a best advantages compared to traditional DPC technique. Matlab software was used to implement the proposed control and compare it with the traditional strategy. This proposed strategy has the downside of using power estimation. In addition to not knowing the number of rules needed to be used to obtain good results, as if a larger number of rules is used, the system becomes slow and this is undesirable. In^56^, a discrete SMC strategy was proposed to improve the characteristic of the DPC technique of the AG, where the SVM technique is used to control the rotor inverter. The use of both discrete SMC strategy and SVM strategy makes DPC control more complex, difficult to implement, and expensive. In addition to the presence of a significant number of gains, which makes it difficult to adjust the dynamic response, which is not desirable. The DPC–SVM strategy based on discrete SMC strategy uses the same estimation equations found in the traditional strategy, which creates problems in the event of a malfunction in the system. The proposed control behavior was verified using Matlab software in terms of power quality, reference tracking, robustness, and THD of current compared to the DPC strategy. The simulation results for this strategy showed that the proposed strategy is effective and has outstanding performance in all tests performed compared to the traditional strategy. However, despite this outstanding performance, the problem of energy ripples remains, especially in the case of durability testing. SMC technique based on fractional-order control was proposed to improve the effectiveness and robustness of the DPC technique of the AG–WT system^57^. The fractional-order SMC (FOSMC) strategy is a strategy characterized by high durability, a significant number of gains, difficult to achieve, complex, and expensive compared to some controls such as SMC or SC technique. In this proposed technique, two FOSMC controllers were used to regulate the *P*_*s*_ and *Q*_*s*_ of generator. In addition to using the FOSMC controller, the SVM strategy is used for the purpose of controlling the inverter, as using the SVM strategy increases the degree of complexity and cost of the system, which is undesirable. In addition to using power estimation, which makes the system affected if the machine parameter values change. This proposed strategy was implemented using Matlab software, and showed outstanding performance results compared to the traditional strategy. However, it is noted that ripples remain present in all tests performed with a somewhat high value of THD of current, which is undesirable. In^58^, SC technique is combined with the STA technique to overcome the drawbacks of the DPC technique of the AG-based MRWT system. In this new DPC strategy, a SC-STA control was used for the purpose of controlling *P*_*s*_ and *Q*_*s*_, where the outputs of these two controls are the reference values of the voltage. The latter is used by the PWM strategy to generate the pulses necessary to operate the inverter. This strategy is characterized by a kind of complexity due to the presence of a significant number of gains, which makes it expensive compared to the DPC strategy. The results of DPC–PWM based on SC–STA controllers were compared with the results of the DPC strategy of 1.5 MW AG-based MRWT system, as the results were good if the DPC–PWM strategy based on SC–STA controller was used. The latter was slightly affected in the durability test and this is shown by the high value of power ripples and the value of THD of current, which is undesirable.

In Table 1, the pros and cons of the strategies found in the literature and which were focused on above are mentioned. These negatives and positives gave us a clear picture to complete and achieve this work. So, all the strategies that have been relied upon in the literature contain different positives and negatives, where energy waves, complexity, difficulty of achievement, and the presence of a large number of gains are among the most prominent of these negatives. Simplicity, quick dynamic response, and ease of implementation are among the strongest and most prominent advantages found in some controls, especially linear strategies. Therefore, the focus was on the most prominent negatives and positives to accomplish this work, as the proposed control is based on using the most prominent positives while outweighing the most prominent negatives present in the strategies.

**Table 1:** Disadvantages and advantages of control strategies found in the literature.

	Advantages	Disadvantages
The first control family: Linear control such as DPC and DTC	Fast dynamic response	High current ripples
	Simplicity of the algorithm	High THD value of voltage/current
	Easy to accomplish	Use capacity estimation
	Not expensive	Affected by changing machine parameters
	It can be applied to all systems	Overshoot
	You do not need to know the mathematical model of the machine	SSE
	Simple controllers are used that are not related to the machine parameters	
The second control family: Nonliear control such as SMC, passivity control, and backstepping control	Durability	Chattering problem
	High performance	It is difficult to adjust the control because there are many parameters
	Satisfactory dynamic response	Complexity
	No SSE	Difficult to achieve compared to linear controls
	No overshoot	Expensive
	It gives a better answer than the linear control types	Related to system parameters
	Using PWM or SVM strategies to control the inverter, which allows obtaining unchangeable frequency	
The third control family: Intelligent control such as NNs, FL, and genetic algorithms	High durability	There are no mathematical rules that facilitate the process of applying these strategies
	Depends on experience	Black box
	Fast dynamic response	You need learning algorithms, as is the case in neural networks
	Doesn’t consume energy	The system becomes slow if a large number of rules (fuzzy logic) or internal layers (NNs) are taken into account
	Easy to apply	
	Easy to accomplish	
	not expensive	
	It is not affected by changing system parameters	
The fourth control family:Hybrid control such as SC-SMC, SC-STA, and BC-SMC	High durability	Complexity
	Good performance	Difficult to apply to complex systems
	Effectively improve system characteristics	Expensive
	Satisfactory dynamic response	Difficult to accomplish
		Having a large number of gains
		Difficulty adjusting the dynamic response
		In some cases you need to know the mathematical form of the system

## Research gap and motivation

All of the above strategies have improved WP system performance by reducing power and current ripples and eliminating the main problem with the high value of THD of current nature of the traditional DFOC system. However, there is still a need to improve current quality and reduce *P*_*s*_ and *Q*_*s*_ ripples, especially in the event of a system fault. On the other hand, it is noted that all the solutions proposed and mentioned above to improve the performance of the DFOC strategy increase the degree of complexity and difficulty of implementation, as these proposed solutions do not lead to a significant increase in the quality of the stream. It is also noted that all the proposed solutions are noticeably affected in the case of the robustness test, which makes us constantly search for a control strategy that has a distinctive performance in the robustness test. Additionally, durability, ease, and simplicity are some of the most emphatic features found in any reliable controller. Therefore, several different points must be relied upon before implementing or completing any system and controlling it, as these points can be identified as follows:

- The degree of complexity of the system is one of the features that must be paid attention to, as the simpler the system is, the easier it is to control, easy to implement, low in cost, low in maintenance, and has a quick dynamic response. In addition to not consuming much energy.
- A small number of parameters makes the control easy to adjust and the dynamic response can be changed simply.
- Distinctive performance is one of the most prominent tasks that must be present in any control system or strategy in different working conditions.
- Control robustness necessarily leads to system robustness. Therefore, it is necessary to use a control strategy that does not depend on the mathematical model of the system, which allows better results to be given in the event of a change in the machine parameters or a temporary failure of the device.

In power generation systems, power ripples, current quality, and durability are among the most undesirable negatives, and their impact increases if the machine parameters change, especially in the case of using control strategies that rely on the mathematical model such as BC technique, passivity control, and predictive control. These strategies provide unsatisfactory results if the machine parameters change, making the system likely to crash. All of the strategies mentioned above cannot give excellent performance and overcome the problem of low power and current quality. Therefore, attention must be paid to searching for other control strategies that are characterized by simplicity, ease of implementation and application, easy to adjust, inexpensive, high durability, high performance, and great effectiveness in improving the system’s characteristics.

Hence, in this work, a new nonlinear strategy is proposed to control the power generation system and reduce the current and power ripples significantly with a constant switching frequency. The proposed work permit is a new form of control for the WP generation system using an AG type generator, with the aim of reducing the costs of the energy consumption and production bill and obtaining high quality of current/energy. Accordingly, the control proposed in this work depends on the combination of various control strategies to obtain distinctive performance, as this proposed control will provide an appropriate solution and a new outlook in the field of control. In short, the proposed control is an integration of the advantages of four different strategies with the aim of obtaining a different and new control that is characterized by high efficiency in improving the quality of the current and the generation system itself. The works mentioned above gave a great incentive to search for new strategies that have several advantages, and this is based on a new idea that relies on combining various existing strategies to be used to obtain a new control with high performance, high durability, and great efficiency in improving the characteristics of the system. Therefore, it was proposed to combine four different strategies as the best solution that can be relied upon to overcome the problem of power ripples, durability, and low current quality, especially in the event of a change in the machine parameters.

### Challenges

The simplicity of the power generation system is of great importance in reducing the costs of energy production and consumption, as structural simplicity makes the control strategy have lower costs and a lower degree of complexity. Therefore, the control strategy greatly affects the operational performance of the system, both positively and negatively, which makes the use, implementation, and selection of this strategy one of the most important priorities that must be considered before proposing any WP generation system.

The type of turbine is among the challenges that were dealt with, as a regular turbine was used instead of an MRWT to maintain the simplicity of the system, reduce the cost of energy production and consumption, ease of implementing and implementing the system, and reduce periodic maintenance. As is known, using an MRWT requires more regular maintenance than a regular turbine. In addition, choosing the type of control used to control the AG was among the challenges we faced in completing this work, as we focused on simplicity, durability, high performance, ease of implementation, and ease of adjustment. Dynamic response is one of the most important challenges we have faced. Therefore, the strategy of integrating various controls was relied upon, using controls that have all the specifications mentioned above. Another challenge faced in this completed work is how to calculate control parameters to obtain distinctive performance and high efficiency. Therefore, there were many possible solutions from reliable artificial intelligence strategies, such as genetic algorithms, grey wolf optimization, and ant colony form. However, PSO technique was chosen as a suitable solution in this work to calculate the parameters of the proposed strategy. The advantage of the proposed controller is that it relies on simple strategies for its configuration, as it uses both the PI and SC technique. In addition to using the PSO technique to determine the controller parameter values. Therefore, this work gives a new picture of strategies that do not exist in the field of scientific research. In addition, the proposed strategy is not related to the system parameters, which will make the proposed power system perform distinctively, especially in the case of changing machine parameters. Moreover, having a small number of parameters will make the dynamic response easy to adjust and thus the performance of the control system is high, resulting in smoothness, which is very acceptable.

Despite the many advantages of the proposed strategy, this strategy also faces some challenges that may limit its adoption, as these challenges lie in:

- The type of intelligent strategy that should be used to obtain control gain values in order to obtain good results.
- *Strategy structure* Determining the structure of the control strategy may pose a challenge to obtain better results because the FOPI and FOSC controls can be used in series or in parallel.
- *Decreased power/current quality* The proposed strategy relies on power estimation, which may cause a decline in power/current quality if the machine parameters change.
- *High number of gains* Using three or four different strategies (PI control, SC technique, PSO technique, fractional-order control) to configure the proposed controller will make the proposed control contain a significant number of gains and thus poses the problem of determining their values. In addition to what is the smart strategy that gives values to the gains for distinguished performance in terms of reducing energy ripples and increasing the quality of the current.

Despite these challenges, the completion of this work and the realization of the proposed control technique for a 1.5 MW AG-based WT has generated significant interest in developing control strategies. This work and efforts aim to overcome challenges and launch a series of other highly efficient solutions for controlling machines. In addition to unleashing the full potential of this promising strategy with the potential to improve it in the future.

## Contribution

The defects and problems that were identified in the above-mentioned works, which were the reason for presenting this work, depend on the use of an appropriate solution that has distinctive performance on the power generation system studied. The proposed control was based on the traditional strategy based on PI controller and some published scientific works.

This paper presents an improvement of the advantages of the traditional DFOC technique of an 1.5 MWAG-based WT using the fractional-order synergetic control fractional-order PI (FOSC–FOPI) controller based on PSO algorithm under different working conditions of the system such as the occurrence of a malfunction in the machine. So the FOSC–FOPI controller is the main contribution of this paper. This type of controller was presented for the first time in this work to improve the characteristics of the DFOC technique, and it depends on the use of four different strategies (SC technique, fractional-order control, PI controller and PSO algorithm) in the concept and idea to obtain a control scheme characterized by high efficiency.

The PSO algorithm was used to calculate the FOSC–FOPI controller parameters and get better results compared to the traditional PI controller. The proposed FOSC–FOPI–PSO controller is a simple, easy implementation controller and robust compared to the traditional PI controller. The second contribution of the paper is the use of the FOSC–FOPI–PSO controller in order to improve the performance and overcome the problems of the DFOC technique of 1.5 MW AG-based variable-speed WT system.

The point of the proposed DFOC–FOSC–FOPI–PSO technique is to control the *P*_*s*_ and *Q*_*s*_ of the 1.5 MW AG and improve the quality of the network current during the change of system parameters and simplify the control of generators. Main objectives of the proposed DFOC–FOSC–FOPI–PSO technique is to reduce the *P*_*s*_ and *Q*_*s*_ ripples, maintaining a DC constant link voltage, minimize the THD value of current, reduce the overshoot and SSE values, and generally enhance the durability of the generating system. The designed system generates high quality current from WP while changing the parameters of the system. The Matlab program is used for the purpose of implementing the designed system and studying the extent of performance and efficiency of the proposed control compared to traditional control in terms of graphical and numerical results in the case of various tests. The two methods were compared in terms of tracking references, robustness, and reduction rates for rice time, overshoot, power ripples, SSE, response time, and current ripples. In addition to reducing the value of THD of current, the numerical and graphical results included in various tests demonstrated the performance, efficiency, efficiency, and high durability of the designed technology compared to traditional control.

The proposed strategy provided excellent graphical and numerical results compared to traditional control. These results can be summarized in the following points:

- Improved characteristics of the DFOC technique of the 1.5 MW AG.
- A numerical comparison was made to determine the efficiency and characteristics of the designed nonlinear technique compared to the traditional DFOC technique based on PI controllers under various working conditions.
- Significant increase in system durability.
- Reducing the THD value of the current by high ratios (99.16%, 71.26%, and 96.57%) in all tests compared to the traditional DFOC technique.
- Minimized current and energy ripples of the 1.5 MW AG compared to the DFOC technique based on PI controller.
- Improved response time of the *P*_*s*_ and *Q*_*s*_.
- Reducing rice time, SSE, and overshoot values of the *P*_*s*_ and *Q*_*s*_ by high percentages.

### Paper organization

The article is divided into 6 different interconnected sections intended to clarify the objectives of the work undertaken. In “Model of WT system” section summarizes WT system. A designed FOSC–FOPI–PSO controller for improving the performance of the DFOC technique of a AG–WT system is introduced in “Design of the FOSC–FOPI–PSO controller” section. The design of DFOC technique based on FOSC–FOPI–PSO controllers is given in “DFOC–FOSC–FOPI–PSO technique” section. The comparative results of simulation studies of the efficiency of the designed DFOC–FOSC–FOPI–PSO strategy are introduced in “Numerical results” section. As result, conclusions are given in “Conclusions” section.

## Model of WT system

**Figure 1:**
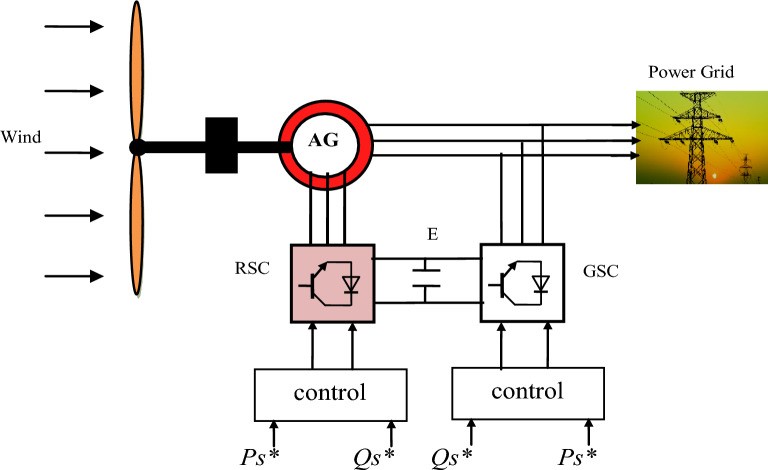
Block diagram of the WT system.

The WT system consists of a power electronics, turbine, filtre, control systems, generator (AG), and a transformer, as seen in Fig. 1. This system is used to convert WP into electrical power^9^. The latter is exploited in several different fields, such as public lighting and the fed of electrical appliances. Any generator can be used in this generation system, where the generator is selected according to its characteristics and features^58^. AGs are widely used compared to other generator types due to their superior characteristics, such as a variable WS and the robustness^59^. To feed the rotor part of the AG, two inverters of the same number of IGBT are used, where the first inverter converts alternating current into continuous current and the second inverter converts continuous current into alternating current. Inverters are used in this technologyto control the rotational speed of the generator.

### WT system characteristics

The mechanical energy produced by WP is related to WS (*V*), tip speed ratio (***λ***), and power coefficient (C_p_), which can be expressed by the following equation^9^:

1 $$ P_{m} = \frac{1}{2}\rho R^{2} C_{p} \left( {\beta , \lambda } \right)V^{3} $$

where, *R* is the radius of the turbine (m), *β* is the blade pitch angle (degrees), and *ρ* is the air density (kg/m^3^).

The C_p_ is very important and it is a nonlinear function, its value is related to both λ and blade pitch angle (β). This coefficient can be calculated by Eq. (2). C_p_ is the maximum power value that can be obtained from wind-by-WT, when λ is at optimal value (λ_op_).

2 $$ C_{p} = \left( {0.5 - 0.167} \right)\left( {\beta - 2} \right) \times \sin \left( {\frac{{\pi \left( {\lambda - 0.1} \right)}}{{18.5 - 0.3\left( {\beta - 2} \right)}}} \right) - 0.0018 \times \left( {\beta - 3} \right)\left( {\beta - 2} \right) $$

Equation (3) represents the λ of the WT system. Through the equation, the λ is greatly affected by the *V* and the rotational speed (*Ω*_*t*_) of the generator^9^.

3 $$ \lambda = \frac{{R \cdot {\Omega }_{t} }}{V} $$

### Mathematical model of AG

Traditionally, AG is one of the most prominent AGs that were used in the field of WP because of its multiple characteristics, where durability and ease of control are among the most prominent characteristics of this machine. In addition, the speed of this machine can be controlled by feeding the rotor with less voltage than the stator voltage, which gives an advantage not found in other machines. Accordingly, the output power sent to the network can be controlled by controlling the inverter that feeds the moving part of the machine.

The AG flux, and voltage equations are illustrated in Eq. (4)^9^, where *R*_*s*_ and *R*_*r*_ represent stator and rotor resistance, *L*_*r*_ and *L*_*s*_ rotor and stator inductance, *Ψ*_*dr*_ and *Ψ*_*qr*_ are the rotor flux linkages, *V*_*dr*_ and *V*_*qr*_ are the rotor voltage linkages, *Ψ*_*ds*_ and *Ψ*_*qs*_ are the stator flux linkages, *V*_*ds*_ and *V*_*qs*_ are the stator voltage linkages.

4 $$ \left\{ {\begin{array}{*{20}l} {V_{dr} = R_{r} I_{dr} - w_{r} \Psi_{qr} + \frac{d}{dt}\Psi_{dr} } \hfill \\ {V_{qr} = R_{r} I_{qr} + w_{r} \Psi_{dr} + \frac{d}{dt}\Psi_{qr} } \hfill \\ {\Psi_{dr} = L_{r} I_{dr} + MI_{ds} } \hfill \\ {\Psi_{qr} = MI_{qs} + L_{r} I_{qr} } \hfill \\ {V_{ds} = R_{s} I_{ds} - w_{s} \Psi_{qs} + \frac{d}{dt}\Psi_{sd} } \hfill \\ {V_{qs} = R_{s} I_{qs} + w_{s} \Psi_{ds} + \frac{d}{dt}\Psi_{qs} } \hfill \\ {\Psi_{qs} = MI_{qr} + L_{s} I_{qs} } \hfill \\ {\Psi_{ds} = L_{s} I_{ds} + MI_{dr} } \hfill \\ \end{array} } \right. $$

Equation  (5) represents torque (*T*_*e*_) and the relationship that relates torque with speed^53,58^. This equation is used to know the evolution of the AG speed, where *T*_*e*_ represents the electromagnetic torque, *I*_*dr*_ and *I*_*qr*_ are the rotor current linkages, and *Ω* is the speed of the AG.

5 $$ \left\{ {\begin{array}{*{20}l} {T_{e} = 1.5 p \times \frac{M}{{L_{s} }}\left( { - \Psi_{sd} \times I_{rq} + \Psi_{sq} \times I_{rd} } \right)} \hfill \\ {T_{e} = J \times \frac{{d{\Omega }}}{dt} + f \times \Omega + {\text{T}}_{{\text{r}}} } \hfill \\ \end{array} } \right. $$

The *P*_*s*_ and *Q*_*s*_ can be calculated using the Eq. (6).

6 $$ \left\{ {\begin{array}{*{20}l} {Q_{s} = 1.5 \times \left( { - I_{qs} \times V_{ds} + V_{qs} \times I_{ds} } \right)} \hfill \\ {P_{s} = 1.5 \times \left( {V_{qs} \times I_{qs} + I_{ds} \times V_{ds} } \right)} \hfill \\ \end{array} } \right. $$

## Design of the FOSC–FOPI–PSO controller

Both PI controller and SC technique are among the easiest to use and simplest controllers to apply compared to some controllers such as SMC and BC techniques. PI controller is among the most popular in the industrial field due to its low cost of completion and low maintenance, which can be accomplished by the μA741 integrated circuit^36^. The PI controller can be expressed by Eq.  (7), where it has two coefficients K_i_ and K_p_. Using these two coefficients the response of the PI controller is adjusted and improved. On the other hand, the PI controller can be expressed by tranfer function using Eq. (8).

7 $$ w\left( t \right) = K_{p} \cdot e\left( t \right) + K_{i} \cdot \smallint e\left( t \right) \cdot dt $$

where e(t) is the error (S = X* − X), K_i_ and K_p_ are the constant gains.

8 $$ \frac{w\left( s \right)}{{e\left( s \right)}} = \frac{{K_{i} \cdot s + K_{p} }}{s} $$

The SC technique is one of the most simple mathematical models and can be applied to various systems very easily. On the other hand, SC technique is a nonlinear technique characterized by durability, fast dynamic response, and ease of implementation compared to some nonlinear controls such as BC and SMC^60^. On the other hand, fractional-order control is one of the strategies that became famous in the last century for its ability to significantly improve the efficiency of systems, where durability and simplicity of implementation are the most prominent features of this strategy compared to other controls^61^. The use of fractional-order control in WT systems improves electrical current quality and increases system durability compared to similar systems using traditional controllers such as the PI controller^62^.

SC technique is based on error derivation^63^. This control can be easily applied to complex systems compared to the SMC technique. This control technique is shown in Eq. (9).

9 $$ u\left( t \right) = T \cdot \dot{S} + S,\quad T > 0 $$

where *T* is positive gain. Through which the speed of convergence is controlled. *S* is the linear sliding surface. The solution to Eq. (9) is as follows:

10 $$ S\left( t \right) = S_{0} \cdot e^{t/T} $$

In Table 2, the pros and cons of PI controller and SC technique are given. However, the PI controller is less robust compared to SC technique. The parameters of the PI controller are difficult to adjust for a WT system based on high nonlinearity with uncertain operating conditions.

**Table 2:** Comparison between PI and SC technique.

Controller type		Cons	Pros
Controller I	PI controller	Long setting time	Easy to stabilize faster response than just P controller
		It is difficult to adjust the control because there are two parameters	Easy to implement
		Narrower range of stability	No SSE
Controller II	SC technique	SSE	It gives a better answer than the rest of the other types
		Chattering problem	Easy to perform
			Robustness
			It is facile to adjust the control because there is one parameter

The fractional-order control, PI controller, and SC technique are used to control the *P*_*s*_ and *Q*_*s*_ of an AG-based WT. Through these actions, it is not possible to eliminate the power ripples by using these two controls. In addition, the quality of the current remains low, especially when using a PI controller compared to the SC technique^27^. However, In the case of dealing with non-linear values, the fractional-order control has a high efficiency in dealing with these non-linear values compared to the PI controller, as the fractional-order controller has two additional degrees of freedom, which makes it characterized by high performance and makes it a suitable solution for controlling electrical machines such as AG.

As is known, fractional calculus is one of the most prominent mathematical strategies that appeared many years ago in the field of mathematics and was introduced in the field of controlling electrical systems. Fractional calculus or fractional-order control its use leads to effective improvement of systems. With the simplicity that characterizes it, its use does not increase the complexity or cost of completing the system, and this is what makes it one of the most prominent solutions in several applications, as mentioned above. Like any other strategy, the fractional calculus strategy has pros and cons. The cons of this strategy can be highlighted in the following points: (1) Fractional calculus strategies can provide enhanced control performance for complex systems, especially those with non-linear and characteristics. They can offer improved stability and better disturbance rejection compared to traditional controllers, (2) Fractional calculus strategies offer additional degrees of freedom in tuning. They provide more flexibility in adjusting the controller parameters to achieve desired system performance and response characteristics, and (3) Fractional calculus strategies can exhibit increased robustness against parameter variations, external disturbances, and system uncertainties. This property makes them suitable for applications where maintaining stability and performance under varying operating conditions.

Similar to the positives that it enjoys and its distinguished performance, there are negatives to the fractional calculus strategy that limit its spread and use, and this is due to several points that are as follows: (1) Designing and implementing fractional calculus strategies can be more complex compared to traditional controllers, (2) They often require advanced mathematical tools, (3) The complexity involved in the design and implementation of fractional calculus strategies often requires advanced mathematical tools and techniques, making real-time implementation difficult. Additionally, the need to convert fractional calculus strategies to integer-order for real-time application might lead to a decrease in performance, as this conversion process can compromise the controller’s ability to effectively handle complex system dynamics, and (4) Fractional calculus strategies can impose a higher computational burden due to the involvement of complex mathematical operations. This increased computational load may lead to higher hardware requirements, longer processing times, and potential limitations in real-time applications.

Simulating a fractional-order operator “$s^{ - \delta }$ and $s^{\mu }$” is in most cases very complicated. Several techniques for simulating fractional-order operators have been developed in the literature. Most of them are based on approximating the irrational (non-integer order) function of the operator by a rational (integer order) function in the “*s*” domain. These techniques involve calculating the system’s output using an equivalent continuous rational model with a special representation^64,65^. They are called analog or frequency-domain approximations. These methods can be identified in each of the following strategies: (1) Carlson’s technique, (2) Matsuda’s technique, (3) Charef’s technique, (4) EFC (Continuous Fractional Expansion) technique, (5) Oustaloup’s technique, (6) Diffusive technique^66,67^.

So, through these mentioned strategies, the Oustaloup approximation technique was chosen as the best strategy for the fractional calculus to implement the proposed controller to control the capabilities, reduce ripples, and increase the durability of the system. This method is based on the continuous-time approximation of the irrational transfer function of the fractional-order operator $s^{\mu }$ to a rational transfer function. This approximation uses a recursive distribution of N zeros, and N poles lying in the frequency band $[{w}_{b},{w}_{h}]$, The Oustaloup approximation of the fractional operator is given by^68^:

11 $$ G\left( s \right) = s^{\mu } \cong s_{{\left[ {w_{b} ,w_{h} } \right]}}^{\mu } \cong K\mathop \prod \limits_{i = 1}^{N} \frac{{s + w_{i}^{\prime} }}{{s + w_{i} }}, \mu \in {\mathcal{R}} $$

where N denotes the number of recursive poles and zeros.

The gain, poles and zeros can be calculated respectively from:

12 $$ K = Gain = w_{h}^{\mu } $$

13 $$ w_{i}^{\mu } = Zero = w_{b} \left( {\frac{{w_{h} }}{{w_{b} }}} \right)^{{\frac{{i + N + \frac{1}{2}\left( {1 - \mu } \right)}}{2N + 1}}} $$

14 $$ w_{i} = Poles = w_{b} \left( {\frac{{w_{h} }}{{w_{b} }}} \right)^{{\frac{{i + N + \frac{1}{2}\left( {1 + \mu } \right)}}{2N + 1}}} $$

In this section, four different techniques are combined to obtain a highly robust and highly effective technique for improving the advantages of the electric power generation system. These techniques are represented in PI controller, SC technique, fractional-order control, and PSO algorithm. These four techniques were used while maintaining the simplicity that characterizes both PI controller and SC technique. Therefore, a new control scheme to overcome power ripples and improve the current quality of the AG–WT systems. In addition, the performance and efficiency of both the PI controller and SC technique are improved.

The proposed FOSC–FOPI–PSO technique is a new controller with specific characteristics such as robustness and simplicity. Thus, the special characteristics of the designed FOSC–FOPI–PSO technique are used to improve the performance of the AG–WT system.

The proposed control technique is based on a combination of the characteristics of fractional-order control, PI controller, PSO algorithm, and SC technique, where the fractional-order SC (FOSC) and FOPI controller are placed in series to obtain more robust controller while maintaining the simplicity and ease of implementation that characterize them. The basic idea is to make the FOSC controller the error or input of the FOPI controller. On the other hand, the PSO algorithm was used as a better solution for calculating the parameters of the proposed controller because of its characteristics. In other words, the proposed controller is a modification of the traditional PI controller, whereby FOSC controller is used to make the FOPI controller a more robust controller compared to the PI controller while maintaining the simplicity that characterizes the FOPI controller. Among the advantages of this proposed controller are durability, ease of implementation, simplicity, low cost, and fast dynamic response speed. However, among the minuses of this controller is the presence of five coefficients (β, α, K_1_, K_2_, and K_3_) as shown by Eq. (15):

15 $$ y\left( t \right) = \left( {K_{3} \times \frac{{S_{1} }}{{s^{\beta } }} + K_{2} \times S_{1} } \right)\left( {K_{1} \times \frac{{dS^{\alpha } }}{dt} + S} \right) $$

where *S* and* S*_1_ are the errors (*S* is the error between the reference and measured values (S = X* − X)).

Equation (16) expresses *S*_1_, where the value of *S*_1_ is related to the value of* S*.

16 $$ S_{1} = \left( {K_{1} \times \frac{{dS^{\alpha } }}{dt} + S} \right) $$

With:

17 $$ S_{1} = \left( {K_{1} \times \frac{{d(X^{*} - X)^{\alpha } }}{dt} + (X^{*} - X)} \right) $$

where *S*_1_ is the error or surface, X* is the reference value, and *X* is the measured value.

In order to implement the proposed controller, the fractional-order control parameter is taken according to the following values: $N=5$ and $[{w}_{b}={10}^{-4} ,{w}_{h}={10}^{4}]$

The presence of five coefficients makes it difficult to adjust and improve the response to the designed controller. In the proposed controller, the PSO algorithm was used to calculate the parameters of the proposed FOSC–FOPI controller. The PSO technique is among the smart techniques that appeared in the last years of the last century. This algorithm is based on the simulation of social behavior and movement of members in a flock of birds of school of fish searching for food sources. This technique operates as follows: each condidate solution moving through the search space looks for promising regions on the basis of its previous experiences and those of its neighbor particles^69^. Researchers are interested in PSO technique because of its benefits, including its ease of conception and implementation and dependence only on a fitness function.

Numerous studies and tests support the effectiveness of the PSO technique as an optimization approach^70^. Integer programming, minimum–maximum problems, multi-objective optimization problems, and many application issues in actual tasks are just a few examples of the various domains in which it is used. However, due to individual conformity during the communication exchange process between the individual and population during an iterative search, the standard PSO technique may reduce population diversity and limit the solution to particular local optimization points, failing to obtain global optimization^70^. The algorithm works as follows: moving across the search space, each particle (candidate solution) searches for suitable sites based on its previous experience and those of its neighbors. At each iteration, the calculated results are used to calculate the new position of each particle:

18 $$ \left\{ {\begin{array}{*{20}l} {v_{i}^{k + 1} = wv_{i}^{k} + c_{1} r_{1} \left( {Pbest_{i}^{k} - X_{i}^{k} } \right) + c_{2} r_{2} \left( {Gbest_{i}^{k} - X_{i}^{k} } \right)} \hfill \\ {r_{1} , r_{2} \in \left[ {0, 1} \right]\;are\;random\;numbers} \hfill \\ \end{array} } \right. $$

19 $$ X_{i}^{k + 1} = X_{i}^{k} + v_{i}^{k + 1} $$

where*, Gbest*_*i*_^*k*^ is the global optimum solution, and *Pbest*_*i*_^*k*^ is the optimum personal solution.

Table 3 contains the control parameters for the PSO algorithm used to optimize the proposed FOSC–FOPI controllers.

**Table 3:** PSO parameters.

Parameters	Values
Swarm size	50
Max iteration	100
*c*_1_	0.1
*c*_2_	1.2
*w*	0.8

**Figure 2:**
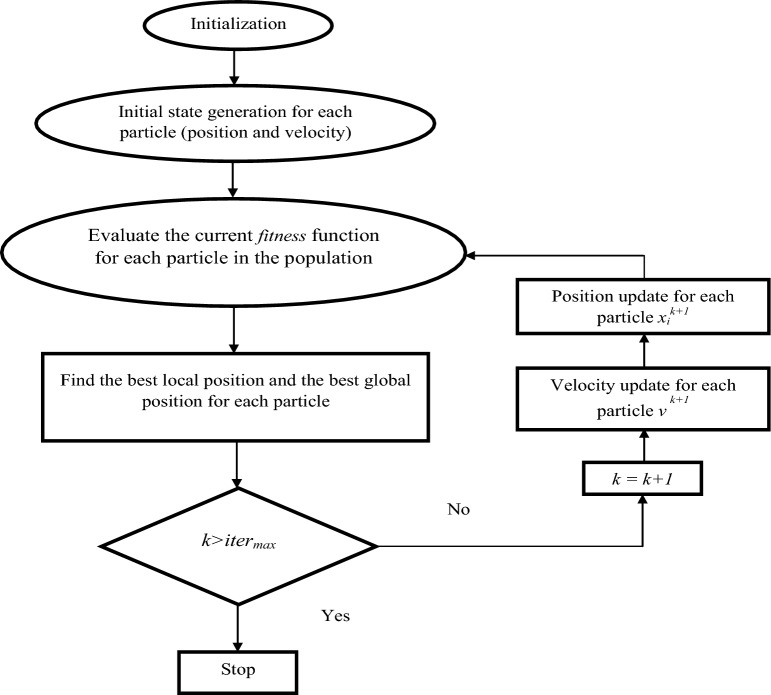
The organigram of PSO algorithm.

The algorithm used to calculate the parameter values of the proposed controller is represented in Fig. 2, as it depends on comparing solutions and finding the best suitable solution that can give satisfactory results. In addition to determining both the best local position and best global position for each particle.

In order to apply the PSO algorithm, several error-related functions are used. The use of these functions is necessary to complete this algorithm to determine the parameter values of the proposed controller. So, to complete this work, we chose to use integral of the absolute error (IAE) to implement the algorithm and calculate the parameters of the proposed controller because of its simplicity and high accuracy. On the other hand, IAE is commonly used as a fitness function in optimization problems, including those involving control system design and tuning. When using the IAE as a fitness function, the goal is typically to minimize the error between the desired output and the actual output of a system.

IAE is relatively easy to calculate and implement, making it a practical choice for fitness functions in optimization algorithms. Its simplicity allows for efficient evaluation during the optimization process. IAE is less sensitive to error values than integral of time-weighted absolute error (ITAE) and innovative training & software expertise (ITSE). It takes into consideration the absolute error without temporal weighting factors, which makes it more robust when the system is subject to errors or significant disturbances.

IAE involves a simple calculation that doesn’t require additional time-weighting or squaring operations like ITAE and ITSE. This simplicity makes it more computationally efficient and easier to implement in optimization algorithms.

To improve this work and give very satisfactory results, IAE was used to sum the absolute errors of the currents and forces together to build the objective function. In this way, the performance and efficiency are well increased and this is shown by the results obtained in the results section. Equation (21) expresses the mathematical form of IAE used in this work in order to determine the gain values of the proposed controller.

20 $$ IAE = \mathop \smallint \limits_{0}^{\infty } \left| {e\left( t \right)} \right|dt = \mathop \smallint \limits_{0}^{\infty } \left( {\left| {\Delta I_{qr} } \right| + \left| {\Delta I_{dr} } \right| + \left| {\Delta P_{s} } \right| + \left| {\Delta Q_{s} } \right|} \right)dt $$

**Figure 3:**
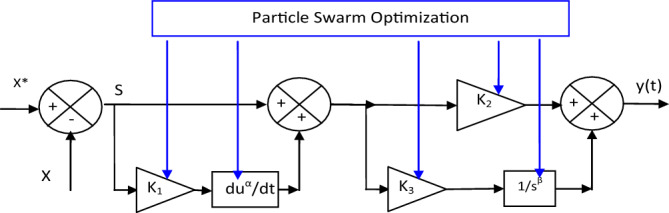
Designed FOSC–FOPI–PSO controller.

Finally, the designed FOSC–FOPI controller based on PSO technique can be expressed by Fig. 3. Four different techniques are used to obtain a simpler controller with high robustness and low implementation cost compared to some techniques such as HOSMC and BC techniques. The use of the PSO algorithm in this proposed controller will increase the durability and significantly improve the dynamic response of this controller, thus reducing the power ripples and increasing the quality of the current generated from the wind system. On the other hand, Fig. 3 gives a clear picture of the degree of complexity of the proposed controller compared to the PI controller, as well as the number of gains present through which the dynamic response is controlled.

So, it can be said that the proposed controller is more complex than the PI controller, which makes it more expensive and energy consuming. In addition to the presence of a large number of gains, which makes it difficult to change and control the dynamic response with ease compared to the PI controller, which contains only two gains. Therefore, the disadvantage of the proposed controller can be determined in both complexity and the number of gains compared to the PI controller.

## DFOC–FOSC–FOPI–PSO technique

The DFOC strategy is one of the most simple and easy to implement strategies. Plus there is a small amount of gain which makes adjusting the dynamic response easier. In this strategy, the PWM technique or SVM technique is used for the purpose of controlling the inverter, where a PI control is used for the purpose of determining reference voltage values. The latter is used for the purpose of generating control pulses. The working principle and concepts of the primacy of DFOC technique and its advantages are available in^71^ and the pros and cons and necessary mathematical relationships for DFOC technique can be found in^72^. Despite the many uses of DFOC technique in controlling generation systems using REs, there are still problems with this strategy. Ripples at the *P*_*s*_ and *Q*_*s*_ level are among the main weaknesses of the DFOC technique^73^. An equally important drawback of ripples that must be addressed is the high value of the THD of current^74^. According to the works^71,72^, the reference values of the voltage in the DFOC are expressed by the Eq. (22).

21 $$ \left\{ {\begin{array}{*{20}l} {V_{dr}^{*} = K_{p} \cdot \left( {\frac{{V_{s} }}{{w_{s} L_{m} }} - i_{dr} - \frac{{L_{s} Q_{s}^{*} }}{{L_{m} V_{s} }}} \right) + K_{i} \cdot \smallint \left( {\frac{{V_{s} }}{{w_{s} L_{m} }} - i_{dr} - \frac{{L_{s} Q_{s}^{*} }}{{L_{m} V_{s} }}} \right) \cdot dt} \hfill \\ {V_{qr}^{*} = K_{p} \cdot \left( { - i_{qr} - \frac{{L_{s} P_{s}^{*} }}{{L_{m} V_{s} }}} \right) + K_{i} \cdot \smallint \left( { - i_{qr} - \frac{{L_{s} P_{s}^{*} }}{{L_{m} V_{s} }}} \right) \cdot dt} \hfill \\ \end{array} } \right. $$

where K_i_ and K_p_ are the gains of the controllers.

Using this strategy in the field of control gives many negatives, especially overshoot/SSE value, as this strategy presents large overshoot/SSE for *P*_*s*_ and *Q*_*s*_. In addition to a high value of THD of current, which makes the quality of the current low, which is undesirable. On the other hand, if the machine parameters are changed, this strategy provides unsatisfactory results, as a significant increase in overshoot/SSE value and a noticeable decrease in the quality of the current are observed. This appears through an increase in the THD value of current.

So the proposed DFOC strategy is the best solution to the defects found in the traditional DFOC-PI technique and some control strategies. The proposed strategy is a type of nonlinear control that uses a FOSC–FOPI–PSO controller to control power, where two controllers are used for the purpose of control. This proposed strategy is different from several existing strategies^54,57^.

In this part, a new strategy for DFOC technique is introduced to overcome the drawbacks of the classical DFOC-PI technique. This proposed DFOC technique is based on the use of both FOSC–FOPI–PSO and PWM technique, where two FOSC–FOPI–PSO techniques are used to regulate the *P*_*s*_ and *Q*_*s*_ of the AG-WT system. However, PWM technique is used to generate IGBT control signals.

This proposed strategy is a modification of the traditional DFOC-PI technique, where the PI controller is eliminated and replaced with the proposed controller, with the aim of increasing performance, efficiency, and efficiency in improving the characteristics of the system in general. Also, the simplicity of the traditional control has been preserved, as the use of PWM technique to control the inverter increases the simplicity of the system and its total cost.

**Figure 4:**
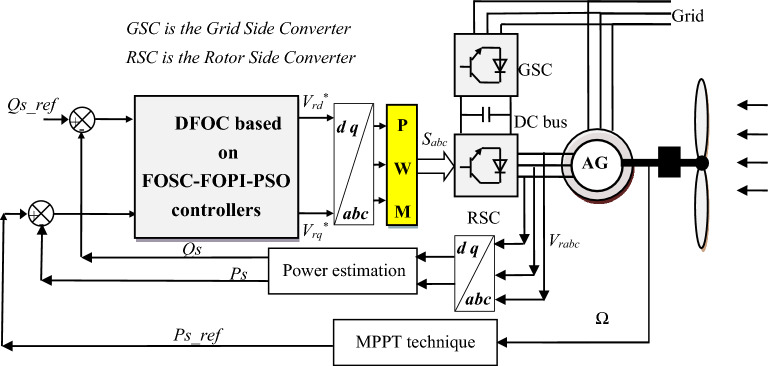
Proposed DFOC techniqueof AG-WTS.

Compared to the DFOC technique, the designed technique is more robust, has many advantages and high efficiency. The proposed structure of the DFOC technique based on the FOSC–FOPI–PSO controllers is given in Fig. 4. The reference direct rotor voltage value (*V*_dr_^*^) of RSC controller is generated from FOSC–FOPI–PSO controller, where the *Q*_*s*_ error is the input of the FOSC–FOPI–PSO controller. The reference quadrature rotor voltage value (*V*_qr_^*^) of RSC controller is generated from the FOSC–FOPI–PSO controller, where the *P*_*s*_ error is the input of this controller.

In this proposed strategy, the MPPT strategy is used to calculate the reference value of the *P*_*s*_. So, the *P*_*s*_ becomes related to the WS, as its value changes according to the change in the WS. In addition, using the MPPT strategy makes the system generate more power than the wind, which is desirable.

In terms of complexity, the proposed strategy is considered more complex than the traditional strategy as a result of the proposed controller being more complex than the PI controller. In addition to the presence of a large number of gains, which makes it difficult to control the dynamic response compared to the traditional strategy. So, the negative of the proposed control can be limited to the complexity and the presence of a significant number of gains.

The *P*_*s*_ and *Q*_*s*_ error signals are derived as:

22 $$ \varepsilon_{Ps} = P_{s}^{*} - P_{s} $$

23 $$ \varepsilon_{Qs} = Q_{s}^{*} - Q_{s} . $$

where *ɛ*_*Ps*_ and *ɛ*_*Qs*_ depict error signals. The outputs of the FOSC–FOPI–PSO technique are expressed by:

24 $$ V_{dr}^{*} = (K_{1} .\frac{{d\left( {\frac{{V_{s} }}{{w_{s} L_{m} }} - i_{dr} - \frac{{L_{s} Q_{s}^{*} }}{{L_{m} V_{s} }}} \right)^{\alpha } }}{{dt^{\alpha } }} + \left( {\frac{{V_{s} }}{{w_{s} L_{m} }} - i_{dr} - \frac{{L_{s} Q_{s}^{*} }}{{L_{m} V_{s} }}} \right))K_{2} + K_{3} \mathop \smallint \limits_{0}^{t} \left( {\frac{{V_{s} }}{{w_{s} L_{m} }} - i_{dr} - \frac{{L_{s} Q_{s}^{*} }}{{L_{m} V_{s} }}} \right)^{\beta } dt^{\beta } $$

25 $$ V_{qr}^{*} = (K_{1} .\frac{{d\left( { - \frac{{L_{s} }}{{V_{s} L_{m} }}P_{s}^{*} - I_{qr} } \right)^{\alpha } }}{{dt^{\alpha } }} + \left( { - i_{dr} - \frac{{L_{s} P_{s}^{*} }}{{L_{m} V_{s} }}} \right))K_{2} + K_{3} \mathop \smallint \limits_{0}^{t} \left( { - i_{dr} - \frac{{L_{s} P_{s}^{*} }}{{L_{m} V_{s} }}} \right)^{\beta } dt^{\beta } $$

In this proposed technique, both voltage and current are measured to estimate both the *P*_*s*_ and *Q*_*s*_. We do not need to know the rotational speed of the AG or the position of the rotor in this proposed technique. In this proposed technique, inner loops are not used which makes the dynamic velocity of the *P*_*s*_ and *Q*_*s*_ very fast compared to the DFOC technique and some strategies such as VC technique. To estimate the capabilities, the stator/rotor flux is calculated according to the following equations:

26 $$\left\{\genfrac{}{}{0pt}{}{{\Psi }_{r\beta }={\int }_{0}^{t}({V}_{r}-{R}_{r}\times {i}_{r\beta })dt}{{\Psi }_{r\alpha }={\int }_{0}^{t}({V}_{r}-{R}_{r}\times {i}_{r\alpha })dt}\right.$$

27 $$\left\{\genfrac{}{}{0pt}{}{{\Psi }_{s\beta }={\int }_{0}^{t}({V}_{s}-{R}_{s}\times {i}_{s\beta })dt}{{\Psi }_{s\alpha }={\int }_{0}^{t}({V}_{s}-{R}_{s}\times {i}_{s\alpha })dt}\right.$$

Also, Eq. (28) can be used to calculate the rotor/stator flux from Eqs. (27) and (26).

28 $$\left\{\begin{array}{c}\left|{\Psi }_{r}\right|=\sqrt{\left({\Psi }_{r\beta }^{2}+{\Psi }_{r\alpha }^{2}\right)}\\ \left|{\Psi }_{s}\right|=\sqrt{\left({\Psi }_{s\beta }^{2}+{\Psi }_{s\alpha }^{2}\right)}\end{array}\right.$$

The stator flux in α–β axes can be calculated in another way using the AG parameters, where the following equation can be used:

29 $$\left\{\begin{array}{c}{\Psi }_{s\beta }=\sigma {I}_{r\beta }{L}_{r}\\ {\Psi }_{s\alpha }=\sigma {I}_{r\alpha }{L}_{r}+{\Psi }_{s}\frac{M}{{L}_{s}}\end{array}\right.$$

where, $$\sigma =1-\frac{{M}^{2}}{{L}_{s}{L}_{r}}$$

Finally, the *P*_*s*_/*Q*_*s*_ is estimated using Eq. (30). As is known, the estimation of the powers is necessary to calculate the error in the *P*_*s*_ and *Q*_*s*_ (*ɛ*_*Ps*_ and *ɛ*_*Qs*_).

30 $$\left\{\begin{array}{c}{Q}_{s}=-\frac{3}{2}\left(\frac{{V}_{s}}{{\sigma \times L}_{s}}{\times \Psi }_{\beta r}-\frac{{V}_{s}{\times L}_{m}}{{\sigma {\times L}_{r}\times L}_{s}}\right)\\ {P}_{s}=-\frac{3}{2}{V}_{s}{\times \Psi }_{r\beta }\times \frac{{L}_{m}}{{\sigma \times {L}_{r}\times L}_{s}}\end{array}\right.$$

In Table 4, a look is given at the similarities and differences between the proposed DFOC–FOSC–FOPI–PSO technique and the traditional DFOC technique from several aspects, as the DFOC–FOSC–FOPI–PSO technique is characterized by its robustness and ease of implementation. Moreover, the DFOC–FOSC–FOPI–PSO technique can reduce the ripples of torque, flux, current, and *Q*_*s*_ significantly compared to the DFOC technique.

**Table 4:** A comparative study between the DFOC and DFOC–FOSC–FOPI–PSO strategies.

	DFOC	DFOC–FOSC–FOPI–PSO
HC	Yes	No
Simplicity	Simple	Simple
ST	Yes	No
SSE	High	Low
PWM technique	No	Yes
Quality of power	Low	High
Rise time	High	Low
Robustness	Low	High
MPPT technique	Yes	Yes
*P*_*s*_ and * Q*_*s*_ estimation	Yes	Yes
Degree of complexity	Low	Low
*P*_*s*_ and * Q*_*s*_ ripples	High	Low
FOSC–FOPI–PSO controller	No	Yes
THD	High	Low
Response dynamic	Slow	Quick

## Numerical results

In this section, the proposed DFOC–FOSC–FOPI–PSO technique is achieved using Matlab software, where the DOC–FOSC–FOPI–PSO technique is compared with the traditional DFOC technique from several aspects such as the value and ratios of the current and *P*_*s*_/*Q*_*s*_ ripples, SSE, and THD value. Moreover, two tests are proposed to verify the effectiveness of the DFOC–FOSC–FOPI–PSO compared to the DFOC technique based on PI controllers. The parameters used in this work are the same parameters used in the work^7,23,28^, and these parameters are listed in the Appendix section.

**Figure 5:**
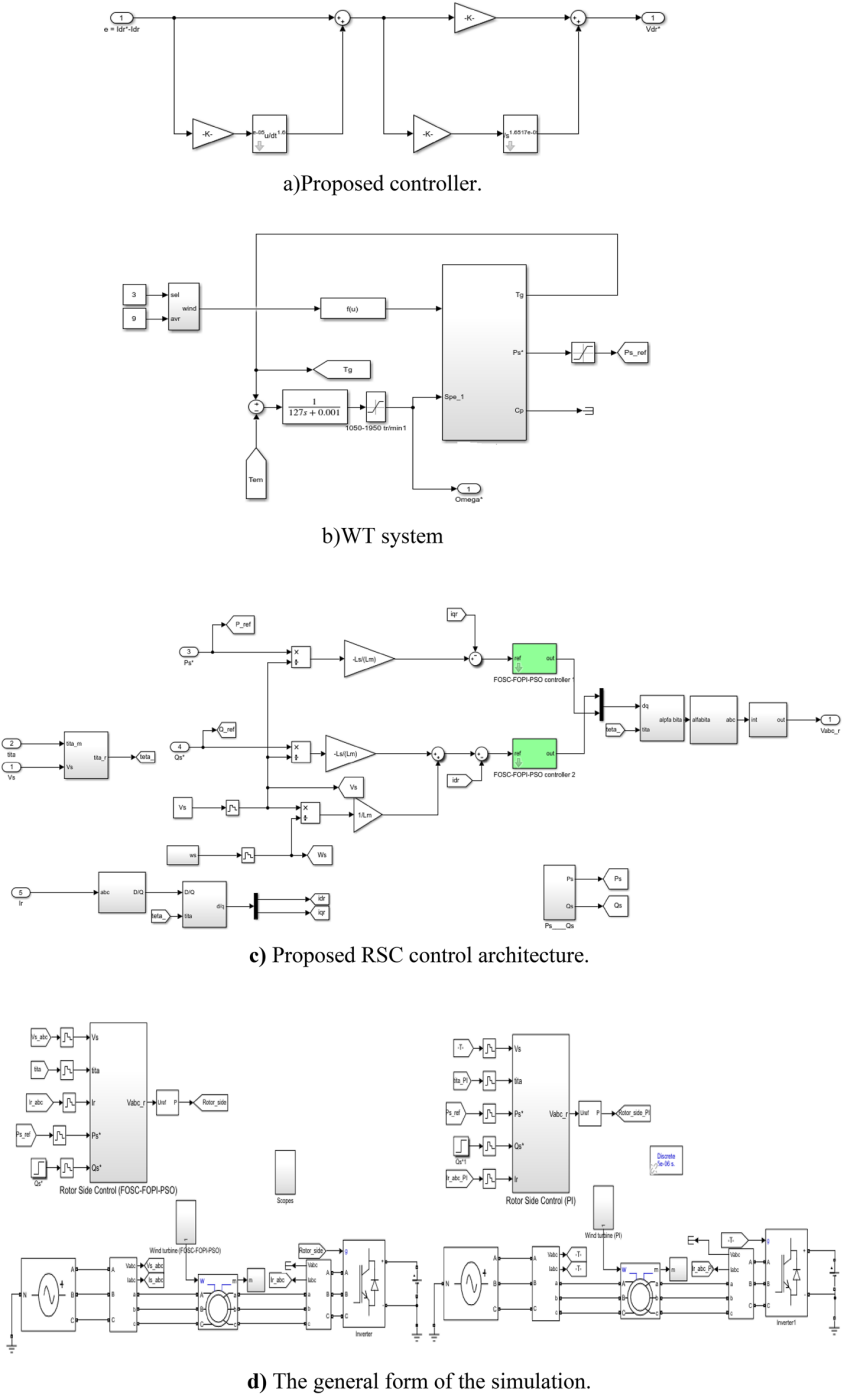
General outline of the strategies.

Figure 5 represents the general outline of the control techniques used in this work for AG control, where the Fig. 5a show the proposed nonlinear controller in Matlab, Fig. 5b show the general form of MRWT in the simulation, Fig. 5c shows the proposed DFOC technique of AG-based WT system and Fig. 5d show the both techniques used in this work.

### First test

**Figure 6:**
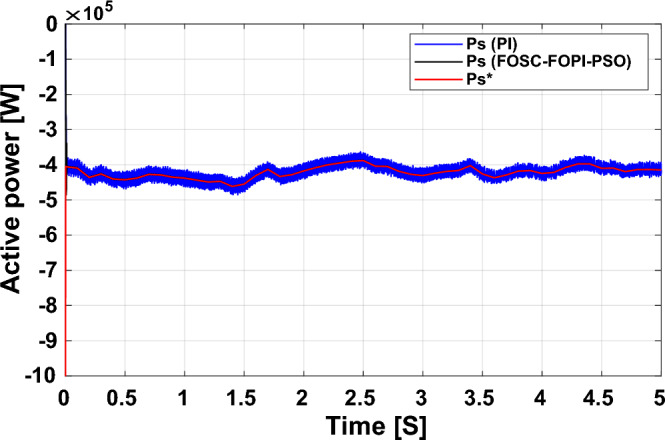
* P*_*s*_.

**Figure 7:**
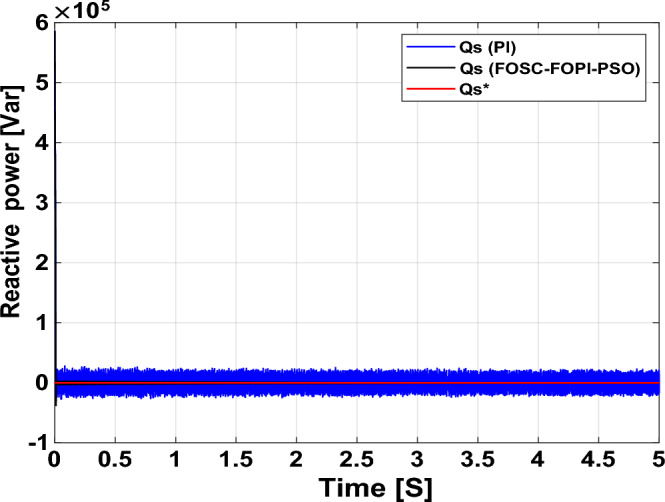
* Q*_*s*_.

**Figure 8:**
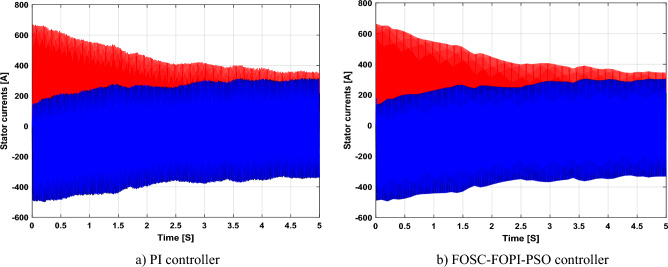
Currents.

**Figure 9:**
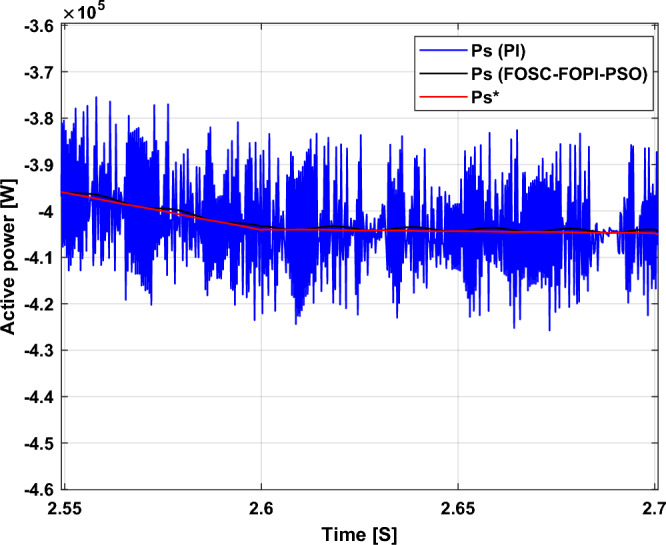
Zoom in *P*_*s*_.

**Figure 10:**
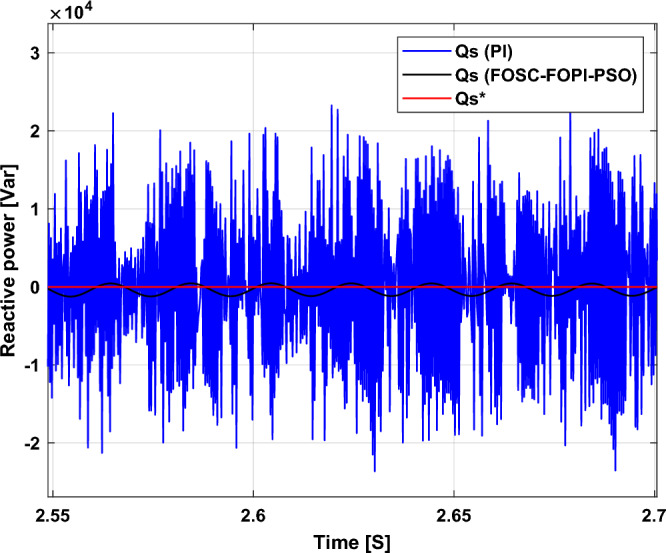
Zoom in *Q*_*s*_.

**Figure 11:**
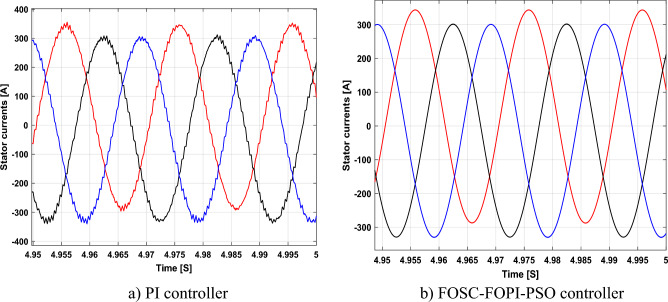
Zoom in currents (FOSC–FOPI–PSO).

**Figure 12:**
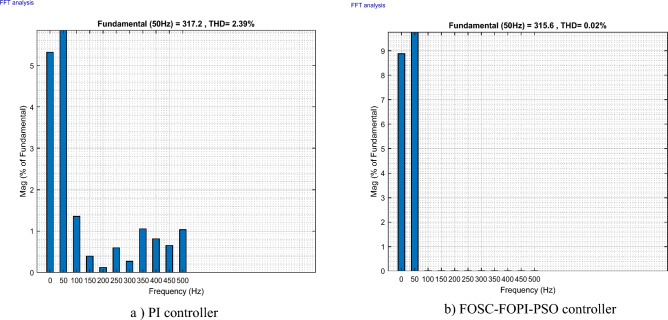
THD value of current.

In this test, the performance of the DFOC–FOSC–FOPI–PSO technique is tested compared to the traditional DFOC technique in the case of using variable WS, where the results obtained are represented in Figs. 6, 7, 8, 9, 10, 11 and 12. Numerical results are also extracted for response time, rice time, overshoot, and SSE of *P*_*s*_ and *Q*_*s*_ of AG-WT system. These numerical results for the two controls are shown in Tables 5 and 6. The numerical results give a clear picture of the superiority and high performance of the controls.

**Table 5:** Comparison of ripple values between the designed and conventional controller.

	*Q*_*s*_ (VAR)	*Ia*(A)	*P*_*s*_ (W)
PI	33,760	17.6	30,700
FOSC–FOPI–PSO	250	0.5	100
Ratios	99.25	97.15%	99.67%

**Table 6:** Values and ratios of the SSE, rise time, overshoot, and response time in the first test case.

	*P*_*s*_ (W)	*Q*_*s*_ (VAR)	
DFOC-PI	Rise time	0.0077	0.00825 s
	Overshoot	9700	19,750
	SSE	17,500	18,210
	Response time	0.00825 s	0.00925 s
Proposed technique	Rise time	0.005	0.008 s
	Overshoot	30,100	25,680
	SSE	500	555
	Response time	0.0055 s	0.0085 s
Ratios	Rise time	35.06%	3.03%
	Overshoot	− 67.77%	− 23.09%
	SSE	97.14%	96.95%
	Response time	33.33%	8.10%

Figures 6 and 7 represent the *P*_*s*_ and *Q*_*s*_, respectively. With these two figures, the *P*_*s*_ and *Q*_*s*_ follow the reference values well. The *P*_*s*_ changes according to the change in WS as a result of using the MPPT technique. Regarding the *Q*_*s*_, its value remains constant and zero during the simulation period, as it is not affected by a change in WS.

Figures 8 represent the electric current produced by the AG-WT system for the two controllers (PI controller and FOSC–FOPI-PSO controller). According to this figure, the form of current change is the same as the change of *P*_*s*_ and WS. However, the value of the current is related to the studied system and the *P*_*s*_ to a large extent. In addition, the current takes a sinusoidal frequency of 50 Hz, with more ripples in the case of classical control. The ripples of *P*_*s*_, current, and *Q*_*s*_ are represented in Figs. 9, 10 and 11 for the two controls. Also, the values and ratios of these ripples are recorded in Table 5. Through Table 5 and Figs. 9, 10 and 11, the proposed control scheme significantly reduced these ripples compared to the DFOC technique. The designed technology has reduced these ripples by a high percentage, as evidence of its high efficiency. These reduction ratios were about 99.67%, 97.15%, and 99.25% for the *P*_*s*_, current, and *Q*_*s*_, respectively. So these high reduction percentages give an image of the superiority of the proposed strategy and its high performance compared to the strategy based on the PI controller.

Figures 12a,b represent the THD value of both control schemes. This value was 2.39% and 0.02% for both the conventional control and the proposed one, respectively. The DPC-FOSCFOPI-PSO technique minimized the THD by about 99.16% compared to the DFOC technique. So, it can be said that the designed DFOC–FOSCFO–PSO technique is better than the DFOC technique in improving the quality of the current, and this thing will be confirmed in the next test. The negative of this strategy lies in the value of the amplitude of the fundamental signal of current, where the DFOC–FOSCFOPI–PSO technique provided a lower amplitude compared to the DFOC technique, where the amplitude value was 317.2 A and 315.6 A for both the DFOC technique and the proposed one, respectively.

Table 6 shows the numerical results for the response time, rice time, overshoot, and SSE of *P*_*s*_ and *Q*_*s*_ for the two controls. Through this table, the DFOC–FOSCFOPI–PSO technique presented good reduction ratios for rise time, SSE, and response time of *P*_*s*_ and *Q*_*s*_ compared to the DFOC technique. Also, the DFOC–FOSCFOPI–PSO technique reduced the rise time, SSE, and response time ratios of *Q*_*s*_ by 3.03%, 96.95%, and 8.10%, respectively, compared to the DFOC technique. Regarding *P*_*s*_, the reduction rates were estimated at 35.06%, 97.14%, and 33.33% for rise time, SSE, and response time, respectively, compared to the DFOC technique. These ratios are very satisfactory, indicating the performance of the proposed control in improving system characteristics compared to the DFOC technique. But DFOC–FOSCFOPI–PSO technique provided unsatisfactory results for the overshoot value of both *P*_*s*_ and *Q*_*s*_, where the DFOC technique provided better results with reduction rates estimated at 67.77% and 23.09% for both the *P*_*s*_ and *Q*_*s*_, respectively, compared to the DFOC-FOSCFOPI-PSO technique.

### Second test

**Figure 13:**
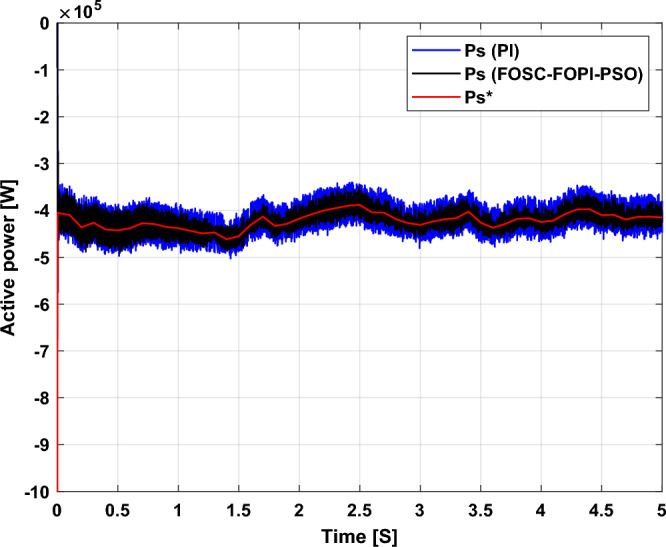
* P*_*s*_.

**Figure 14:**
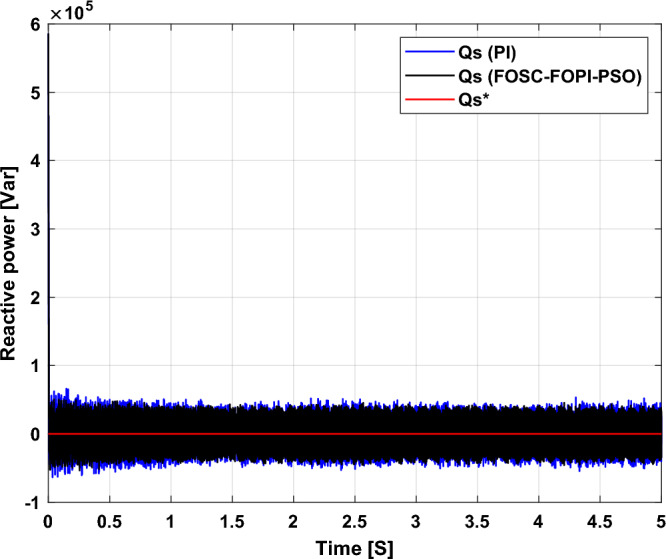
* Q*_*s*_.

**Figure 15:**
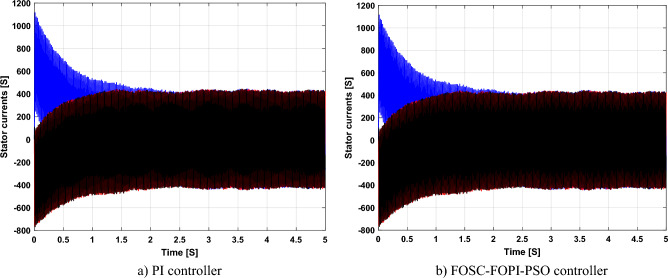
Currents.

**Figure 16:**
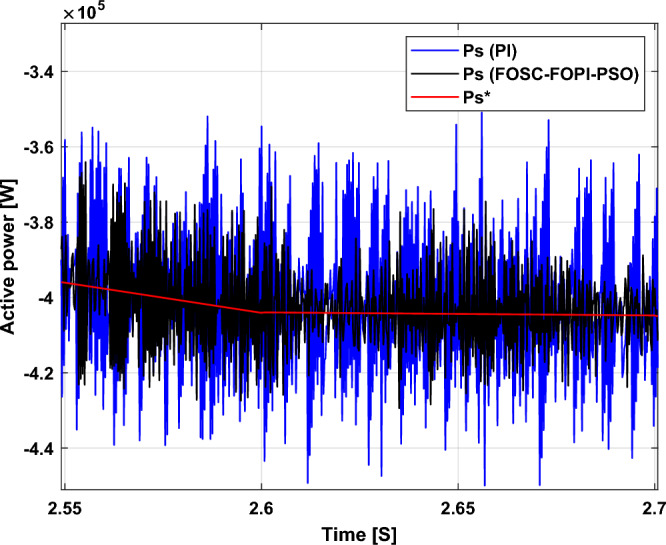
Zoom in *P*_*s*_.

**Figure 17:**
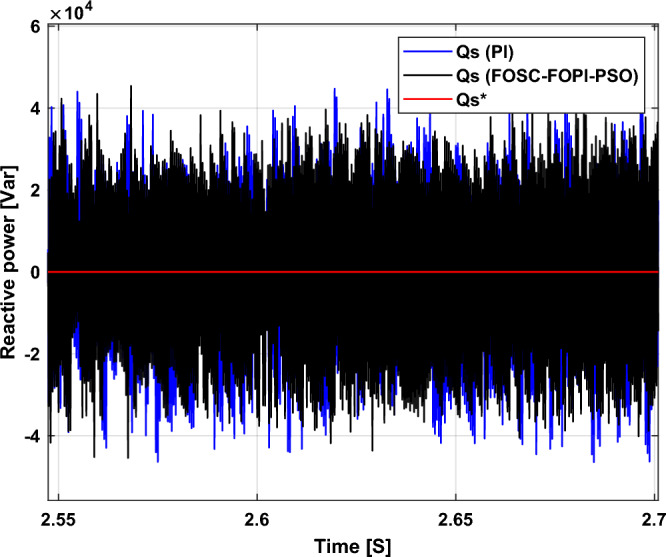
Zoom in *Q*_*s*_.

**Figure 18:**
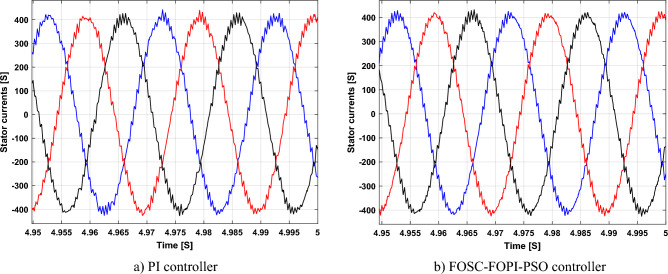
Zoom in currents.

**Figure 19:**
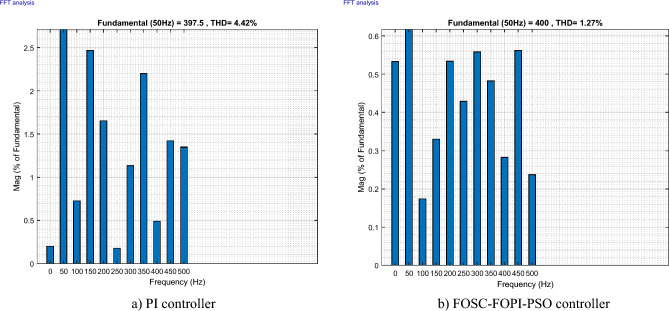
THD values.

This test is to study the behavior of the designed DFOC control scheme if the parameters of the AG are changed. In this test, the generated parameters are changed according to Table 7. This test is proposed to study the robustness of the designed DFOC technique versus the classical technique. The results of this test are represented in Figs. 13, 14, 15, 16, 17, 18 and 19. The numerical results of this test are also extracted for ripple, response time, SSE, and overshoot. These numerical results are reported in Tables 7 and 8.

**Table 7:** New values for the AG parameters.

	*R*_*s*_	*L*_*s*_	*L*_*m*_	*R*_*r*_	*L*_*r*_
New values	0.024 Ω	0.00685 H	0.00675 H	0.042 Ω	0.0068 H
Old values	0.012 Ω	0.0137 H	0.0135 H	0.021 Ω	0.0136 H

**Table 8:** Comparison of ripple values between both controllers (second test).

	*Ia* (A)	*Q*_*s*_ (VAR)	*P*_*s*_ (W)
PI	41	55,780	63,100
FOSC–FOPI–PSO	19	25,260	34,900
Ratios	53.65%	54.71%	44.69%

The *P*_*s*_ and *Q*_*s*_ are shown in Figs. 13 and 14, respectively. Despite the change of AG parameters, the *P*_*s*_ and *Q*_*s*_ follow the references acceptably with preference to the designed control scheme in terms of dynamic response. Also, there is a noticeable effect of changing parameters on the two controls and this is shown by the presence of ripples. This effect is significant in the case of the DFOC-PI technique compared to the designed control scheme. In addition, the *Q*_*s*_ remains constant and zero and is not affected by a change in WS. The value of the *P*_*s*_ is affected by the WS because using the MPPT technique to calculate the reference value of the *P*_*s*_.

The stator currents of both controls are shown in Figs. 15a,b. The current remain in the form of *P*_*s*_, whereby the higher the *P*_*s*_, the greater the value of both current and torque. Despite changing the AG parameters, the current remains sinusoidal with a frequency of 50 Hz, with an advantage over the DFOC-FOSCFOPI-PSO technique in terms of the degree of influence.

Figures 16 through Fig. 18 represent zoom in the *P*_*s*_, *Q*_*s*_, and current for both controls. Through these figures, it can be said that the DFOC-FOSCFOPI-PSO technique is better than the DFOC–PI technique in terms of the degree of influence. This effect appears in the very high ripple values in the case of the DFOC technique compared to the DFOC–FOSCFOPI–PSO technique. The values of these ripples are recorded in Table 8. Through this table, the DFOC-FOSCFOPI-PSO technique minimized the values of the ripples of *P*_*s*_, current and *Q*_*s*_ by good proportions compared to the DFOC-PI technique. These ratios were about 54.71%, 53.65%, and 44.69% for each of the *P*_*s*_, current, and *Q*_*s*_.

The current THD was 4.42% for the DFOC technique and 1.27% for the DFOC-FOSCFOPI-PSO technique (Figs. 19a,b). So the proposed control scheme minimized the THD value by 71.26% compared to the DFOC-PI technique. In addition, despite the change in the AG parameters, the proposed strategy was able to give a value for the amplitude of the fundamental signal of current greater than the amplitude in the case of the DFOC technique, where the value of the amplitude was 397.5 A and 400 A for both the DFOC technique and the proposed one, respectively.

The proposed control provided good numerical results compared to the DFOC technique in terms of rise time, SSE, and response time of *P*_*s*_ and *Q*_*s*_ (Table 9). And response time at rates of 35.06%, 97.14%, and 33.33%, respectively, compared to the DFOC technique. In addition, the reduction ratios for rise time, SSE, and response time for the *P*_*s*_ were 3.03%, 96.95%, and 8.10%, respectively, compared to the DFOC technique, as these ratios indicate the ability of the DFOC-FOSCFOPI-PSO technique to improve the system characteristics. However, in the case of overshoot the *P*_*s*_, the DFOC–FOSCFOPI–PSO technique provided unsatisfactory results, as the DFOC technique gave better values for exceeding compared to the proposed control, and this is shown by the estimated reduction ratio of 30.23%.

**Table 9:** Ratios and values of overshoot, SSE, rise time, and response time of *P*_*s*_ and *Q*_*s*_ in the second test case.

	*P*_*s*_ (W)	*Q*_*s*_ (VAR)	
DFOC-PI	Rise time	0.00325 s	0.0027 s
	Overshoot	26,300	26,090
	SSE	42,800	37,860
	Response time	0.0045 s	0.005 s
Proposed technique	Rise time	0.00275 s	0.0022 s
	Overshoot	37,700	9486
	SSE	29,200	21,150
	Response time	0.003 s	0.0045 s
Ratios	Rise time	15.38%	18.51%
	Overshoot	− 30.23%	63.83%
	SSE	31.77%	44.13%
	Response time	33.33%	10%

With regard to overshoot the *Q*_*s*_, the DFOC–FOSCFOPI–PSO technique presented a good percentage compared to the DFOC technique, as the percentage of reduction was estimated at 63.83% compared to the DFOC technique (Table 9). Through these obtained results, it can be said that the DFOC–FOSCFOPI–PSO technique is the best solution for controlling the AG-WTS because of its ease of implementation, simplicity, and the results obtained. So, through the second and first tests, the override can be considered to be the negativity present in the proposed control, as this negativity is undesirable. This negative can be overcome by using another smart strategy instead of using the PSO technique, as grey wolf optimization can be used for this purpose.

## Steps WS test

**Figure 20:**
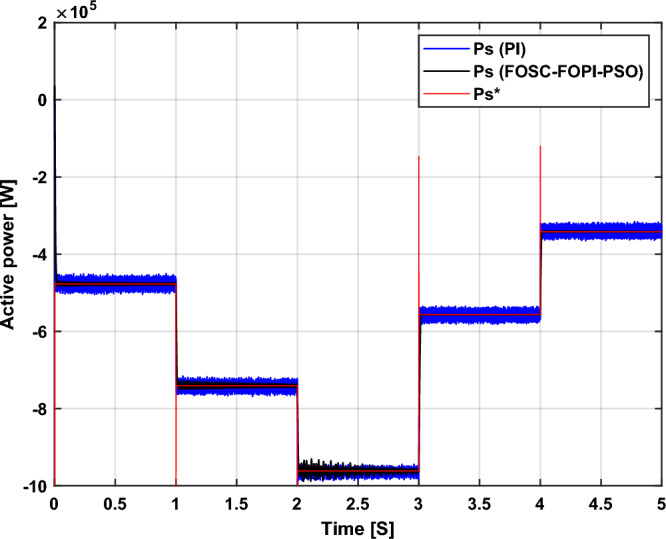
*P*_*s*_.

**Figure 21:**
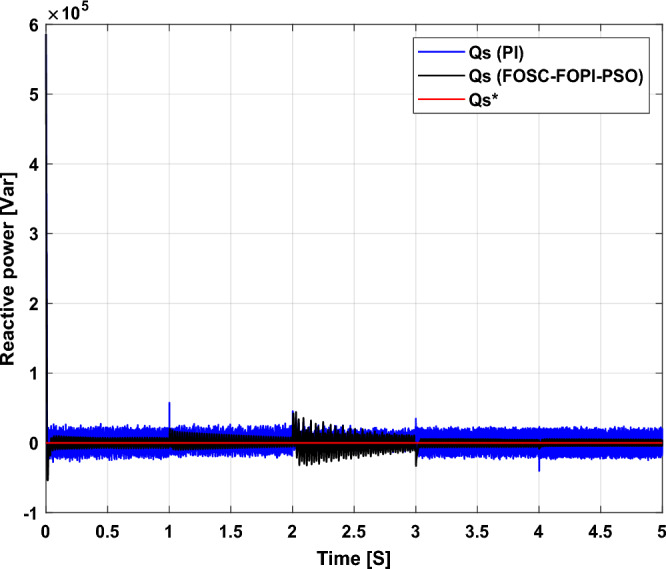
*Q*_*s*_.

**Figure 22:**
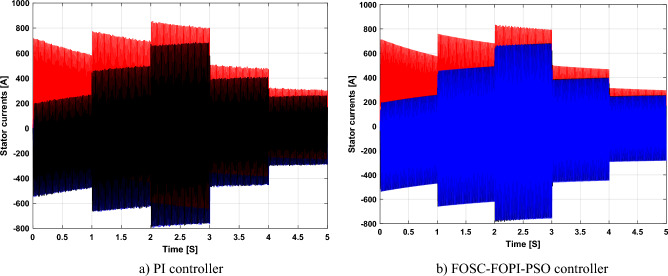
Currents.

**Figure 23:**
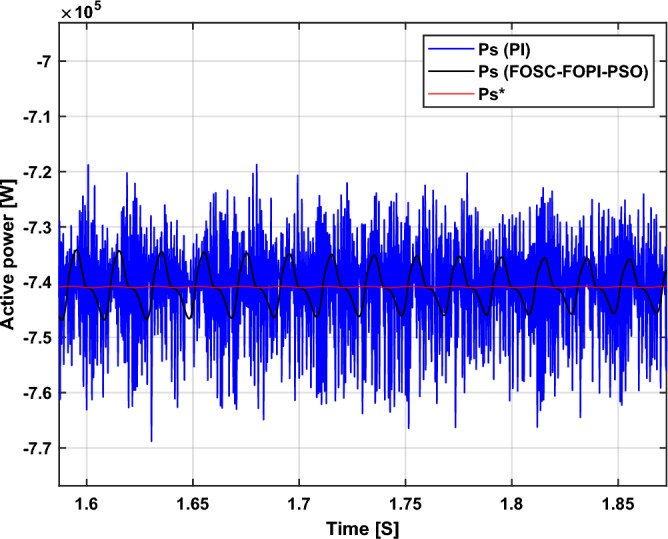
Zoom in *P*_*s*_.

**Figure 24:**
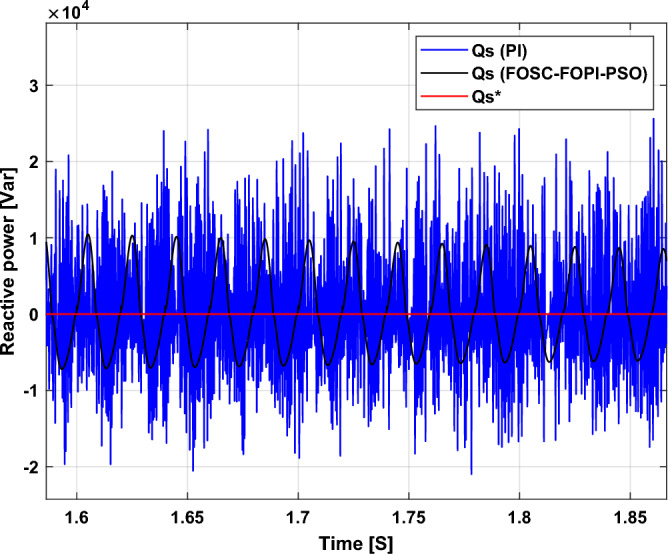
Zoom in *Q*_*s*_.

**Figure 25:**
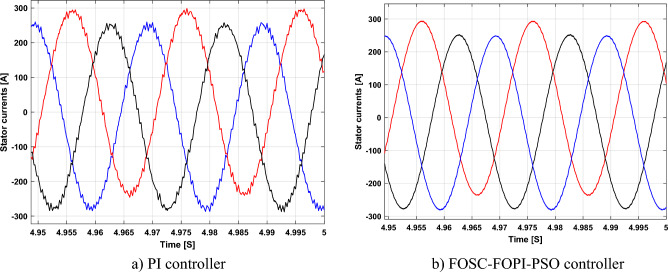
Zoom in currents.

**Figure 26:**
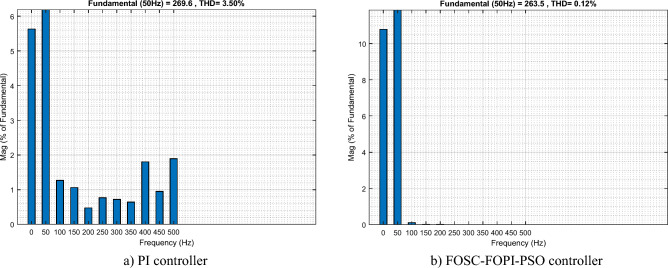
THD values.

This test differs from the previous two tests in terms of the shape of the WS. The latter is in the form of steps, where the DFOC–FOSC–FOPI–PSO technique behavior is studied when the wind speed is in this form compared to the DFOC technique. The numerical and graphical results are shown in Tables 10 and 11 as well as in Figs. 20, 21, 22, 23, 24, 25 and 26. Figures 20 and 21 represent the *P*_*s*_ and *Q*_*s*_ of the two controls, respectively. In this Figures, the *P*_*s*_ and *Q*_*s*_ of the two controllers follow the references well with larger ripples if the DFOC technique is used, where the *Q*_*s*_ takes a zero value and is not affected by the change of the WS (Fig. 21). The *P*_*s*_ represented in Fig. 20 follows the reference well for the two controls, with large ripples at the DFOC level compared to the DFOC–FOSC–FOPI–PSO technique. However, the *P*_*s*_ changes according to the change of WS with less ripples in the case of using the DFOC-FOSCFOPI-PSO technique (Fig. 20). In addition, it is observed that there is a large exceedance in the reference value of *P*_*s*_ if the DFOC technique is used compared to the DFOC–FOSC–FOPI–PSO technique, as this exceedance was at time moments 1 s, 3 s, and 4 s with the exceedance values being 100 KW, 200 KW, 100 KW respectively. This exceedance is the result of a sudden change in the reference value of *P*_*s*_, where traditional control is more affected by this sudden change. So, these values indicate the negativity found in the traditional DFOC strategy compared to the proposed strategy, as the proposed DFOC technique provided excellent results in terms of exceedance values, and this is proven by the graphical and numerical results.

**Table 10:** Ratios and values of ripples.

	*Q*_*s*_ (VAR)	*Ia* (A)	*P*_*s*_ (W)
PI	26,480	25.7	19,100
FOSC–FOPI–PSO	7809	0.9	200
Ratios	70.50%	96.49%	98.95%

**Table 11:** Ratios and values of the rise time, SSE, overshoot, and response time in the third test case.

	*P*_*s*_ (W)	*Q*_*s*_ (VAR)	
DFOC-PI	Rise time	0.0076 s	0.0085
	Overshoot	19,800	9935
	SSE	15,900	21,920
	Response time	0.00875 s	0.009 s
Proposed technique	Rise time	0.0125 s	0.008
	Overshoot	6400	22,220
	SSE	100	4976
	Response time	0.0215 s	0.0085 s
Ratios	Rise time	− 39.20%	5.88%
	Overshoot	67.67%	− 55.28%
	SSE	99.37%	77.29%
	Response time	− 59.53%	5.55%

The values and percentages of ripple reduction for *P*_*s*_*,* current, and *Q*_*s*_ for the two controls are listed in Table 10. Through this table, the DFOC-FOSCFOPI-PSO technique reduced the ripples of the current, *P*_*s*_ and *Q*_*s*_ by rates estimated at 96.49%, 98.95% and 70.50%, respectively, compared to the DFOC technique. These percentages indicate that the efficiency of power and current is high when using the proposed control compared to the traditional control, which is a good thing.

Figure 22 represents the current for the two controls, where the current takes the form of WS change with a sinusoidal shape in the case of the two controls with high quality in the case of the DFOC–FOSCFOPI–PSO technique compared to the DFOC with PI controllers. In Figs. 23 and 24, the DFOC–FOSCFOPI–PSO technique gives less ripples than the DPC technique of *P*_*s*_ and *Q*_*s*_. Also, the current ripples are low in the case of the DFOC–FOSCFOPI–PSO technique compared to the DFOC technique and this is shown in Fig. 25. The latter shows graphically that the current shape is better if the proposed control is used compared to DPC technique based on PI controllers.

The THD value of current for the two techniques is represented in Fig. 26. In Fig. 26, the THD value was 3.50% and 0.12% for both the DFOC with PI controllers and the DFOC-FOSCFOPI-PSO technique, respectively. The DFOC–FOSCFOPI–PSO technique has to reduce the value of THD of current compared to the DFOC technique by an estimated rate of 96.57%. So it can be said that the current quality is very high in the case of the DFOC-FOSCFOPI-PSO technique compared to the DFOC technique. On the other hand, the value of the signal amplitude fundamental (50 Hz) of current for both techniques can be extracted from Fig. 26. This amplitude was 269.6 A and 263.5 A for DPC and DFOC–FOSC–FOPI–PSO technique, respectively. So, it can be said that the DFOC–FOSC–FOPI–PSO technique strategy provided a lower amplitude for the signal amplitude fundamental (50Hz) of current compared to the DFOC strategy, which is undesirable.

The numerical values obtained from this test are given in Table 11. In this table, the response time, overshoot, rise time, and SSE of *P*_*s*_ and *Q*_*s*_ of AG-based WT for the two controls are recorded. Through the table, the DFOC technique provided better results than the proposed control in terms of response time of *P*_*s*_, overshoot of *Q*_*s*_, and rise time of *P*_*s*_ and this is shown through the calculated ratios, where the DFOC technique reduced the values of each of the response time and rise time of *P*_*s*_ by rates estimated at 59.53% and 39.20%, respectively, compared to the proposed control. The same for overshoot value of *Q*_*s*_, where the ratio was estimated at 55.28% compared to the DFOC-FOSCFOPI-PSO technique. These percentages represent the negativity of the proposed control in this test. This negativity can be overcome by using grey wolf optimization because it is more efficient than the PSO strategy. But, the Table 11 shows that the DFOC–FOSCFOPI–PSO technique provided better results than the DFOC technique in terms of response time and rise time of *Q*_*s*_. Also, in terms of overshoot of *P*_*s*_ compared to DFOC technique. These ratios were 5.55% and 5.88% for the rise time and response time, respectively, compared to the DFOC technique. In addition, the DFOC-FOSCFOPI-PSO technique reduced the value of SSE of *P*_*s*_ by an estimated rate of 67.67% compared to the DFOC technique, which is a large percentage and of great importance in the field of control. Regarding the value of SSE, the proposed strategy reduced this value by good percentages compared to traditional control, as the reduction percentages were estimated at 99.37% and 77.29% for both *P*_*s*_ and *Q*_*s*_, respectively, compared to traditional control. So, the DFOC-FOSCFOPI-PSO technique has its pros and cons, like any other control. These drawbacks can be overcome by using another highly efficient algorithm instead of using the PSO algorithm, where grey wolf optimization can be used for this purpose. In addition, the combination of NNs and PSO technique can be used as a suitable and best solution to increase the efficiency and performance of the proposed control.

In Table 12, the results of the first test are compared with the results of the third test in terms of the value of THD of current and amplitude of fundamental (50 Hz) signal of current, where the extent to which the values of amplitude and THD are affected by the change in the shape of the wind speed is given. From this table, it is noted that changing the shape of the wind speed affected the amplitude and THD values in the case of both controls, where the values increased significantly. The percentage of change in values in the two tests is greater in the case of the proposed strategy compared to the traditional strategy, which indicates that changing the shape of the wind speed has a significant impact on the proposed strategy and this is shown through the calculated percentages.

**Table 12:** Comparing the change in the values and ratios of the amplitude of the fundamental (50 Hz) signal and the THD of the current with the change in the shape of the WS.

	THD value		Fundamental signal amplitude (A)	
	DFOC-PI	Proposed technique	DFOC-PI	Proposed technique
First test	2.39	0.02	317.20	315.60
Third test	3.50	0.12	269.6	263.50
Ratios	31.71%	83.33%	15%	16.50%

The study carried out in Table 12 is carried out with energy ripples, where the change in ripple values is studied according to the change in the shape of the WS. The results of the first and third tests are used for this purpose, and the percentage changes in the ripple values between the two tests are listed in Table 13. From this table, it is noted that the power and current ripples are affected by the change in the shape of the WS. In the traditional strategy, it is noted that the power ripples were high in the first test compared to the third test, where the percentage increase was estimated at 37.78% and 76.86% for both *P*_*s*_ and *Q*_*s*_, respectively. However, the current ripples were higher in the third test compared to the first test, where this increase was estimated at 31.51%. These percentages indicate that the effect is very noticeable and is related to the shape of the WS. The proposed strategy is the opposite of the traditional strategy, as it is noted that the power and current ripples are high in the third test compared to the first test. The aspect ratio is estimated at 50%, 96.77%, and 44.44% for *P*_*s*_, *Q*_*s*_, and current, respectively. Therefore, the shape of the WS significantly affected the ripples resulting from the proposed strategy. On the other hand, it is noted that the calculated percentages are high in the case of the proposed strategy compared to the traditional strategy. So, it can be said that the proposed strategy is more affected by the change in the shape of the WS, and this despite the fact that the results of the introduction were better than the traditional strategy.

**Table 13:** Studying the change of energy waves in the first and third tests.

	Traditional technique			Proposed technique		
	*P*_*s*_ (W)	*Ias* (A)	*Q*_*s*_ (VAR)	*P*_*s*_ (W)	*Ias* (A)	*Q*_*s*_ (VAR)
First test	30,700	17.60	33,760	100	0.50	250
Third test	19,100	25.70	7809	200	0.90	7809
Ratios	37.78%	31.51%	76.86%	50%	44.44%	96.77%

Finally, a comparative study between the results obtained from the DFOC–FOSCFOPI–PSO technique with the results of published research in terms of THD value of current and the percentage of ripple reduction for each of the *Q*_*s*_, current and *P*_*s*_. The results of the comparison are recorded in Tables 14 and 15, where Table 14 gives the comparison values in terms of THD value and Table 15 represents the comparison values in terms of the ratios of ripple reduction. Through these two tables, the DFOC–FOSCFOPI–PSO technique in this paper is one of the best and the best of them in efficiency and ability to minimize power fluctuations and improve the quality of the current. Moreover, the proposed control technique provided the lowest value for THD compared to several works completed, which makes it the best control technique that can be proposed for controlling wind systems.

**Table 14:** Comparison in terms of THD value of stator current.

	Strategies		THD (%)
^75^	Indirect FOC technique		6.5
^50^	DPC		8.87
	N-DPC		2.91
	NF-DPC		2.72
^76^	DPC-STA		1.66
^77^	Power control	Technique 1	5.6817
		Technique 2	3.1873
^78^	12 sectors DPC		0.40
^79^	DTC		6.70
	Fuzzy DTC		2.40
^80^	DTC		7.83
	Neural DTC		3.26
^81^	Second-order SMC		3.13
^33^	DTC using L-filter		10.79
	DPC using LCL-filter		4.05
^46^	Integral SMC		9.71
	Multi-resonant-based SMC		3.14
^40^	2-level DTC		8.75
	3-level DTC		1.57
^82^	GA-least squares wavelet SVM		3.39
^83^	DPC-IP		0.43
^84^	DVC-SSMC		0.50
^85^	FOC		3.7
^86^	DPC		6.64
	Modified fuzzy-DPC		3.9
^87^	DPC-PI-PSO		15.70
DFOC–FOSC–FOPI–PSO	0.02

**Table 15:** Comparison in terms of ripple reduction ratios.

Methods		Ratios		
		*Ias* ripples	*P*_*s*_ ripples (%)	*Q*_*s*_ ripples (%)
	Proposed technique	97.15%	99.67	99.25
^50^	DPC with neural algorithm	67.79%	45.26	66.29
	DPC with neuro-fuzzy algorithm	69.33%	57.74	67.13
^51^	STA	3.58%	7.35	2.01
	Modified STA	0.21%	13.44	8.96
^86^	Modified fuzzy-DPC	–	63.79	71.53
^88^	Backstepping control	65%	28.57	46.93
^89^	Intelligent control	63.75%	36	35
^90^	Feedback PI control	59.25%	96.65	37.14
^91^	DPC-SPI-GA	81%	75.98	70.21
^92^	DPC-PD(1 + PI)	–	46.68	47.50
^93^	FOSTA	75.60%	64.34	53.56

Table 16 represents a comparison between the DFOC–FOSCFOPI–PSO technique and some strategies in terms of SSE of *P*_*s*_ and *Q*_*s*_, where appropriate and calculated reduction percentages are recorded in the works. These ratios prove that the DFOC–FOSCFOPI–PSO technique is better than several existing control strategies in terms of the SSE value of *P*_*s*_ and *Q*_*s*_, and this confirms the results recorded in Tables 14 and 15. Also, these ratios confirm that the DFOC–FOSCFOPI–PSO technique has high and reliable performance in the control field. And in renewable energies in particular.

**Table 16:** Comparison in terms of SSE reduction rates.

References		Ratios	
		*Q*_*s*_ (VAR) (%)	*P*_*s*_ (W) (%)
^90^	Feedback PI controller	45.48	78
^88^	Backstepping control	82.70	50
^89^	Intelligent control	35.48	62
^92^	DPC-PD(1 + PI)	78.44	45.83
^91^	DPC-SPI-GA	75.98	83.33
	Designed strategy	99.37	77.29

In Table 17, another comparison was made with some strategies in terms of response time for active and reactive power, where the response time obtained in the second test was taken for comparison. So, from this table, it can be said that the proposed nonlinear strategy is better than several controllers such as DPC technique based on genetic algorithm, DPC, and DPC based on neural PI controllers. This comparison gives us a clear picture that the proposed control is better in terms of the dynamic response of the systems, as it can be relied upon in the future to control machines.

**Table 17:** Comparison in terms of response time for *P*_*s*_ and *Q*_*s*_*.*

References		Time Response (ms)	
		*P*_*s*_ (W)	*Q*_*s*_ (VAR)
^94^	DPC	17 ms	18 ms
	Nonlinear DPC strategy	9 ms	5 ms
^95^		33.8 ms	34.5 ms
^96^		32 ms	–
^97^		15 ms	80 ms
^98^		–	28 ms
^89^	PI-GA	3.4 ms	4.53 ms
	PI-NN	4.52 ms	26.26 ms
Designed nonlinear techinque	Second test	3 ms	4.50 ms

## Conclusions

This work proposes a FOSC–FOPI–PSO controller for the DFOC strategy of AG–WT system. To regulate the AG power and improve the quality of current generated by the AG–WT system and to improve the performance of the traditional DFOC strategy. The DFOC–FOSC–FOPI–PSO can not only effectively weaken the effect of power ripples in the WT system, but also raise the robustness of the system to generate WE and obtain a high quality electric current of the 1.5 MW AG system in various tests such as the robustness tests. The Matlab program was used to implement the proposed strategy, using several tests to study its behavior. The results obtained demonstrated the effectiveness of the proposed nonlinear technique in improving the characteristics of the energy system compared to several other controls. The main novelty and contribution of our work can be summarized in the following points:

- The DFOC–FOSC–FOPI–PSO technique is considered more efficient and effective than the DFOC technique in all proposed tests.
- Improve the characteristics of the DFOC–PI technique of variable-speed WT system driven AG under different working conditions.
- The DFOC–FOSC–FOPI–PSO technique reduced the rice time (The reduction ratios for *Q*_*s*_ were estimated at 3.03%, 18.51%, and 5.88% in the three tests), steady-state error (The reduction ratios for *P*_*s*_ were estimated at 97.14%, 91.77%, and 99.37% in the three tests), response time (The reduction ratios for *Q*_*s*_ were estimated at 8.10%, 10%, and 5.55% in the three tests) and ripples of the *P*_*s*_ (99.67%, 44.69%, and 98.95%) and *Q*_*s*_ (99.25%, 54.71%, and 70.50%) compared to the DFOC-PI technique.
- Using a FOSC–FOPI–PSO technique leads to a lowering of the THD value of the stator current of AGs (the reduction percentage was 99.16%, 71.26%, and 96.57% in the proposed tests).
- The proposed DFOC–FOSC–FOPI–PSO technique is more robust compared to the DFOC-PI strategy.

On other hand, a comparison with some scientific works was also presented to highlight the superiority and need to use the DFOC–FOSC–FOPI–PSO to control systems, as the comparison showed the superiority of the DFOC–FOSC–FOPI–PSO over several strategies in terms of the value of THD of current, ripple reduction ratio, response time, and SSE of AG power. However, the designed technique has a negative represented by the fundamental signal (50 Hz) amplitude in the case of the first test, as this negative is undesirable and can be overcome in the future by using other algorithms such as grey wolf optimization instead of using the PSO algorithm, and there is a wide future scope in applying strategies artificial intelligence (FL or NNs) to increase the robustness, performance, and efficiency of the designed technique.

## Data Availability

Data available on request from the authors. The datasets used and/or analysed during the current study available from the corresponding author on reasonable request. In the event of communication, the first author (Habib Benbouhenni, E-mail: habib.benbouenni@nisantasi.edu.tr) will respond to any inquiry or request.

## Appendix

In Tables 18, 19 and 20, the energy system parameters used for the purpose of carrying out this work and verifying the validity and behavior of the proposed strategy are presented.

**Table 18:** Wind turbine parameters.

Number of blades	3
*R*	35.25 m
*G*	90
*J*	1000 kg m^2^
*f*_*v*_	0.0024 N m s^−1^
*V*_*d*_	4 m/s
*V*_*m*_	25 m/s

**Table 19:** AG parameters.

${P}_{n}$	1500 KW
${V}_{s}$	398/690 V
${I}_{n}$	1900 A
*f*	50 Hz
${L}_{s}$	13.7 mH
${L}_{r}$	13.6 mH
*M*	13.5 mH
${R}_{s}$	12 mΩ
${R}_{r}$	21 mΩ
*p*	2
*J*	1 Mg m^2^

**Table 20:** Grid link settings.

${\omega }_{s}$	314 rad/s
${R}_{g}$	0.002 Ω
${L}_{g}$	0.005 H
C_DC_	4.4 mF

## Abbreviations

DFOC: Direct filed-oriented control
AG: Asynchronous generator
DPC: Direct power control
MPPT: Maximum power point tracking
VSWTS: Variable-speed wind turbine system
PI: Proportional-integral controller
DTC: Direct torque control
FOC: Fractional-order control
SC: Synergetic control
HOSMC: High-order sliding mode control
MSVM: Modified space vector modulation
PSO: Particle swarm optimization
WP: Wind power
FOSC: Fractional-order synergetic control
FOPI: Fractional-order proportional-integral controller
WTS: Wind turbine system
MRWT: Multi-rotor wind turbine
WS: Wind speed
PWM: Pulse width modulation
*P*_*s*_: Active power
*Q*_*s*_: Reactive power
WT: Wind turbine
GA: Genetic algorithm
NN: Neural network
RE: Renewable energy
